# Pre-breeding to establish *Fhb7* in winter wheat using marker-assisted, modified backcrossing

**DOI:** 10.3389/fpls.2026.1863042

**Published:** 2026-08-26

**Authors:** Bhanu Dangi, Kripa Rijal, Bradley Bisek, Francois Marais

**Affiliations:** Department of Plant Sciences, North Dakota State University, Fargo, ND, United States

**Keywords:** disease resistance, *Fhb7*, *Fusarium* head blight, gene introgression, gene pyramiding

## Abstract

Fusarium head blight (FHB) is a destructive disease of wheat (*Triticum aestivum* L.) that causes major grain yield and quality losses. Improving host resistance remains an important strategy for managing FHB; however, resistance is quantitatively inherited and is often strengthened by combining multiple complementary resistance loci. The recently released wheat–*Thinopyrum elongatum* translocation line WGC002 carries *Fhb7* in a Chinese Spring-derived spring wheat background, but this resistance source has not been established in the hard winter wheat (HWW) germplasm. The objective of this study was to introgress *Fhb7* into diverse HWW backgrounds using marker-assisted modified backcrossing while simultaneously selecting for additional FHB resistance loci. WGC002 was crossed with HWW germplasm, and three modified backcrosses were made using seven genetically diverse HWW genotypes. The recurrent and backcross parents contributed additional resistance sources, including *Fhb1*, *Qfhb.rwg-5A.1*, *Qfhb.rwg-5A.2*, and native background resistance from ND Noreen. A total of 322 BC_3_F_2_ plants were evaluated for resistance-marker alleles, Type II FHB disease severity, and plant phenotype, from which 32 BC_3_F_2_ families were selected for further evaluation. Twenty-two segregating BC_3_F_2_:_3_ families were re-tested for marker genotype and agrotype, resulting in the selection of 94 BC_3_F_2_:_4_ families carrying different resistance–locus combinations involving two or more of *Fhb1*, *Fhb7*, *Qfhb.rwg-5A.1*, and *Qfhb.rwg-5A.2*. The remaining 10 selected families were homozygous for one or more resistance–locus combinations, including *Fhb7* alone, *Fhb7* plus *Qfhb.rwg-5A.1*, *Fhb7* plus *Qfhb.rwg-5A.2*, and *Qfhb.rwg-5A.1* plus *Qfhb.rwg-5A.2*, and showed strong Type II resistance. Overall, this study developed diverse HWW pre-breeding materials carrying *Fhb7* alone or in combination with additional FHB resistance loci. These selections provide promising pre-breeding candidates for future HWW breeding efforts, aiming to enhance FHB resistance by pyramiding complementary resistance sources.

## Introduction

Fusarium head blight (FHB), caused primarily by the fungus *Fusarium graminearum* Schwabe, can cause severe yield losses and reduce grain quality in wheat (*Triticum aestivum* L.). Infected grain may accumulate deoxynivalenol (DON), a mycotoxin that is harmful to humans and animals and further reduces the market value of the crop (Lilleboe, 2019; Salgado et al., 2015; Alisaac and Mahlein, 2023). Winter wheat cultivars grown in North Dakota range from very susceptible to only moderately resistant to FHB. There is therefore a critical need to improve the host resistance for reducing FHB-associated losses in hard winter wheat (HWW).

The genetics of FHB resistance is complex and involves numerous quantitative trait loci (QTL) distributed across the genome. Most resistance QTL have small individual effects (Mesterházy et al., 2020). More than 500 FHB-associated QTL have been reported and are clustered into 65 unique physical positions (Buerstmayr et al., 2009), yet no wheat cultivar has been found to be completely resistant to FHB (Bai et al., 2018). The overall FHB resistance level is determined by the additive and interactive effects of multiple QTL (Fuentes et al., 2005).

Breeding research is often focused on pyramiding resistance loci, applying marker-assisted breeding methods. This led to an ongoing search for new and diverse resistance QTL that will make the development of resistant cultivars more effective. *Fhb1*, a major-effect resistance QTL located on chromosome 3BS, is widely used in breeding programs and primarily provides Type II resistance by limiting fungal spread within the spike (Cuthbert et al., 2006; Su et al., 2019; Waldron et al., 1999). A reliable diagnostic marker, IFA-FM227, has been associated with the *Fhb1* candidate gene *TaHRC* (Schweiger et al., 2016). Similarly, *Qfhb.rwg-5A.1* and *Qfhb.rwg-5A.2*, identified in PI277012, provide both Type I and Type II resistance and significantly reduce the percentage of Fusarium-damaged kernels and DON accumulation (Chu et al., 2011; Bai et al., 2018). Genetic background FHB resistance QTL were reported in regular HWW breeding populations; these genes produce moderate and intermediate but useful levels of FHB resistance (Neupane et al., 2024) that can reinforce the effect of larger effect QTL such as *Fhb1* (Brar et al., 2019). While genetic background resistance QTL are generally poorly characterized, they can be effective components of resistance pyramids.

Effective resistance QTL not only occurs in the primary gene pool of wheat but also can be acquired from the secondary and tertiary gene pools (Ma et al., 2022). Wheat wild relatives provide additional sources of FHB resistance that can broaden the genetic base of wheat improvement. Among these sources, *Fhb7* is particularly valuable because its molecular function has been characterized. Wang et al. (2020) cloned a highly promising larger effect FHB resistance gene (*Fhb7*) from *Th. elongatum* (tertiary gene pool) and determined that its 846-base pair (bp) sequence encodes a glutathione *S*-transferase (GST) gene that detoxifies the trichothecene toxin to confer resistance to FHB. Although GST-mediated detoxification of DON is a major component of *Fhb7*-mediated resistance, recent evidence indicates that resistance is achieved through a coordinated defense network rather than a single mechanism. Metabolomic analyses showed activation of glutathione metabolism, antioxidant defenses, phenylpropanoid biosynthesis, lignin accumulation, and multiple DON detoxification pathways, which collectively reinforce secondary cell walls, maintain redox homeostasis, and restrict fungal spread within the spike (Fanelli et al., 2023). Wang et al. (2020) also suggested that *Fhb7* may have entered the *Th. elongatum* genome through fungus-to-plant horizontal gene transfer, as *Fhb7* homologs were not detected elsewhere in the plant kingdom but showed high sequence similarity to homologs from endophytic *Epichloë* fungi. They also demonstrated that *Fhb7* introgressions can confer resistance to both FHB and crown rot in diverse wheat backgrounds without an observed yield penalty.

Several *Fhb7* resistance alleles from *Thinopyrum elongatum* and *Th. ponticum* have been introgressed into the wheat D genome via 7D–7E translocations (Guo et al., 2015; Fedak et al., 2021; Ceoloni et al., 2017). In addition, efforts have been made to transfer the *Th. ponticum*-derived *Fhb7* resistance allele into the wheat A genome through a 7A–7E translocation (Forte et al., 2014). More recently, Zhang et al. (2022) transferred a *Th. elongatum*-derived *Fhb7* allele, *Fhb7^The2^* (referred as *Fhb7* from now onwards), into wheat as a 7BS·7BL–7EL translocation in a Chinese Spring-derived spring wheat background. This germplasm was released as WGC002 and provides a useful donor source for marker-assisted introgression because diagnostic markers are available for *Fhb7* (Zhang et al., 2022; Cai et al., 2024). WGC002 is also valuable because the terminal 7EL segment carrying *Fhb7* is not associated with the yellow flour pigment linkage drag reported in some earlier *Thinopyrum-*derived introgressions (Zhang et al., 2022). However, WGC002 is in a spring wheat/Chinese Spring-derived background, and additional pre-breeding is needed to transfer *Fhb7* into agronomically adapted HWW germplasm.

This study was designed to address that pre-breeding gap by using marker-assisted modified backcrossing to introgress *Fhb7* from WGC002 into genetically diverse HWW backgrounds. A second objective was to recover selections carrying *Fhb*7 together with additional FHB resistance loci, including *Fhb1, Qfhb.rwg-5A.1*, *Qfhb.rwg-5A.2*, and background resistance from adapted HWW parents such as ND Noreen.

## Materials and methods

### Plant material

**Figure 1:**
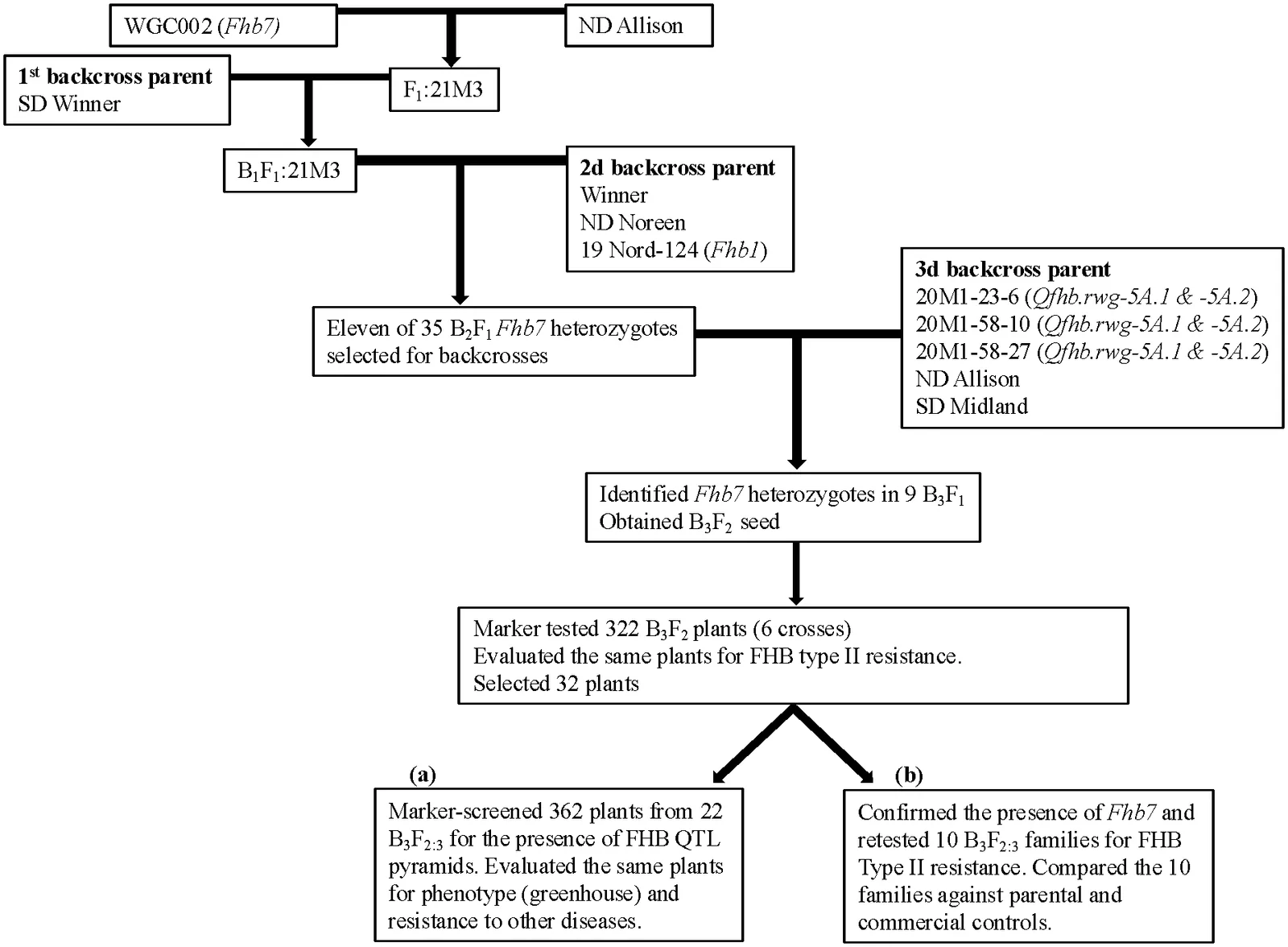
Marker-assisted modified backcrossing scheme used to introgress Fhb7 from WGC002 into hard winter wheat backgrounds. **(a)** Marker-screened 362 plants from 22 BC_3_F_2:3_ families for the presence of FHB QTL pyramids; the same plants were evaluated for phenotype (greenhouse) and resistance to other diseases. **(b)** Confirmed the presence of Fhb7 and retested 10 BC₃F₂:₃ families for FHB Type II resistance; the 10 families were compared against parental and commercial controls.

The overall crossing strategy, parental materials, modified backcrossing scheme, and evaluated populations are summarized in **Figure 1**, and the pedigrees of the nine selected BC_3_F_1_-derived families are provided in **Table 1**. WGC002 was used as the donor of *Fhb7*. Because WGC002 is derived from a Chinese Spring wheat background and is not adapted to HWW production, backcrossing to winter wheat germplasm was used to recover winter wheat adaptation while retaining *Fhb7*.

**Table 1:** Modified backcross pedigrees of nine selected BC_3_F_1_-derived BC_3_F_2_ families used to introgress *Fhb7* from WGC002 into hard winter wheat.

BC_3_F_1_ family^1^	Pedigree	BC_3_F_2_ plants tested^2^	Fully vernalized/FHB-tested plants^3^	Segregating FHB resistance loci
23K468 (p10)	WGC002/ND Allison//2*Winner/3/20M1-23-6	25	22	*Fhb7*, *Qfhb.rwg-5A.1*, *Qfhb.rwg-5A.2*
23K461 (p4)	WGC002/ND Allison//2*Winner/3/20M1-58-27	24	15	*Fhb7*, *Qfhb.rwg-5A.1*, *Qfhb.rwg-5A.2*
23K461 (p5)	WGC002/ND Allison//2*Winner/3/20M1-58-27	24	15	*Fhb7*, *Qfhb.rwg-5A.1*, *Qfhb.rwg-5A.2*
23K461 (p40)	WGC002/ND Allison//2*Winner/3/20M1-58-27	50	22	*Fhb7*, *Qfhb.rwg-5A.1*, *Qfhb.rwg-5A.2*
23K472 (p49)	WGC002/ND Allison//Winner/3/ND Noreen/4/20M1-58-27	24	21	*Fhb7*, *Qfhb.rwg-5A.1*, *Qfhb.rwg-5A.2*
23K472 (p50)	WGC002/ND Allison//Winner/3/ND Noreen/4/20M1-58-27	25	17	*Fhb7*, *Qfhb.rwg-5A.1*, *Qfhb.rwg-5A.2*
23K487 (p67)	WGC002/ND Allison//Winner/3/19Nord-124/4/20M1-58-10	50	21	*Fhb7*, *Fhb1*, *Qfhb.rwg-5A.1*, *Qfhb.rwg-5A.2*
23K466 (p8)	WGC002/ND Allison//2*Winner/3/ND Allison	50	1	*Fhb7*
23K478 (p19)	WGC002/ND Allison//Winner/3/ND Noreen/4/SD Midland	50	11	*Fhb7*
Total		322	145	

^1^Family code followed by plant number in parentheses identifies the selected BC_3_F_1_ plant that was self-pollinated to generate the corresponding BC_3_F_2_ family.

^2^“BC_3_F_2_ plants tested” refers to the number of individual BC_3_F_2_ plants evaluated for marker genotype.

^3^“Fully vernalized/FHB-tested plants” refers to the number of plants that flowered normally and were evaluated for Type II FHB disease severity.

The HWW parents were selected to provide two complementary attributes: agronomic adaptation and additional FHB resistance loci. SD Winner and SD Midland were included because of their favorable agrotype, high yield potential, and intermediate FHB resistance. ND Allison was included because of its high yield potential and putative tolerance to acidic and aluminum-rich soils. ND Noreen was included because of its yield potential, winter hardiness, rust tolerance, and strong background resistance to FHB. The breeding line 19Nord-124 was included because of its favorable agrotype and the presence of *Fhb1*. The advanced breeding lines 20M1-23-6, 20M1-58-10, and 20M1-58–27 were derived from the cross Novus-4/GP80//Monument/3/2*ND Noreen. These lines were selected because marker data indicated that they carried *Qfhb.rwg-5A.1* and *Qfhb.rwg-5A.2*, which were derived from GP80, and because they were expected to retain background resistance from ND Noreen (Ganaparthi et al., 2023). Together, these parents provided a genetic base for developing diverse HWW pre-breeding materials carrying *Fhb7* alone or in combination with other validated FHB resistance loci.

### Modified backcrossing strategy

The term “modified backcrossing” is used here to distinguish this breeding scheme from conventional backcrossing. In a conventional backcrossing program, progeny from a donor parent is repeatedly crossed to the same recurrent parent to recover a single recurrent-parent genome. In the present study, the objective was not to recover one recurrent parent background, but to establish *Fhb7* in multiple adapted HWW genetic backgrounds while preserving genetic diversity and enabling pyramiding with additional FHB resistance loci. Therefore, different HWW parents were used across backcross generations rather than repeatedly backcrossing to only one recurrent parent.

WGC002-derived progenies were advanced through three marker-assisted modified backcrosses to HWW germplasm. WGC002 was first crossed with ND Allison to produce the F_1_ generation. The first backcross was made to SD Winner. The second backcross used three HWW parents: SD Winner, ND Noreen, and 19Nord-124. A total of 35 BC_2_F_1_ plants derived from these crosses were evaluated for agrotype and tested for the presence of *Fhb7*. Eleven *Fhb7*-positive BC_2_F_1_ plants with acceptable agrotype were selected for the third modified backcross. The third backcross used five HWW parents: 20M1-23-6, 20M1-58-10, 20M1-58-27, ND Allison, and SD Midland. These parents were selected to contribute winter adaptation, agronomic performance, and/or additional FHB resistance loci, including *Fhb1*, *Qfhb.rwg-5A.1*, *Qfhb.rwg-5A.2*, and background resistance from ND Noreen-derived germplasm. Following the third backcross, BC_3_F_1_ plants were screened for *Fhb7* and evaluated for agrotype. Nine *Fhb7*-positive BC_3_F_1_ plants derived from six modified backcross pedigrees were self-pollinated to generate BC_3_F_2_ families for marker analysis, Type II FHB resistance testing, and phenotypic selection. The pedigrees of these nine selected BC_3_F_1_-derived families are shown in **Table 1**.

### DNA extraction and marker analyses

Leaf tissues were collected from young plants for DNA extraction. Genomic DNA was extracted at the NDSU HWW laboratory using Invitrogen™ PureLink™ Genomic DNA Mini Kit (Thermo Fisher Scientific) following the manufacturer’s protocol. The USDA-ARS Biosciences Research Lab in Fargo, ND, USA (https://www.ars.usda.gov/plains-area/fargo-nd/etsarc) conducted independent marker analysis for *Qfhb.rwg-5A.1*, *Qfhb.rwg-5A.2*, *Fhb1*, and *Fhb7* using established in-house DNA extraction and marker protocols. The marker Barc186 was used to detect *Qfhb.rwg-5A.1* (Buerstmayr et al., 2009); Gpw2136 was used to detect *Qfhb.rwg-5A.2* (Chu et al., 2011); and IFA-FM227 was used to detect *Fhb1* (Schweiger et al., 2016). For *Fhb7*, the dominant STS marker WGC2315 (Zhang et al., 2022) was used for early-generation screening of BC_1_F_1_, BC_2_F_1_, and BC_3_F_1_ materials because the codominant marker reported by Cai et al. (2024) was not yet available at the time those generations were evaluated. After the codominant marker Xwgc-2320 became available, it was used in the BC_3_F_2_ generation, where segregation analysis required distinction among likely homozygous resistant, heterozygous, and homozygous susceptible marker classes. For selected BC_3_F_2_:_3_ materials, WGC2315 was used as a continuity screen to confirm the presence of the *Fhb7*-associated introgression in progenies derived from previously genotyped BC_3_F_2_ plants. Polymerase chain reaction (PCR) products were separated by electrophoresis on 2% agarose gels in 1× TAE buffer at 80 V for 2.5 h. Gel was stained with ethidium bromide and visualized under ultraviolet light.

### Greenhouse growth conditions and vernalization

Greenhouse evaluations were conducted at Jack Dalrymple Agricultural Research Complex, North Dakota State University. Plants were grown in 5-inch plastic pots filled with a peat-based potting mix containing sphagnum peat moss, perlite, vermiculite, limestone, and a wetting agent. The soil was moistened with a standard nutrient solution prepared from Miracle-Gro^®^ Professional Peat-Lite^®^ Special 20-10–20 according to the manufacturer’s instructions. Seeds were allowed to imbibe for 2 days before vernalization. Plants were vernalized at approximately 39°F for 8 weeks and then transferred to a greenhouse maintained at an average temperature of 70.5°F under a 16-h photoperiod. Supplemental lighting was provided as needed at a photosynthetic photon flux density of approximately 400 µmol m^−2^ s^−1^. After vernalization, slow-release fertilizer Multicote^®^ (6) (15-7-15 + 1.2 Mg + minors) was added to each pot.

### BC_3_F_2_ greenhouse evaluation and selection

An unreplicated greenhouse evaluation was planted in December 2023 to assess 322 BC_3_F_2_ plants derived from the nine selected BC_3_F_1_ plant families (**Table 1**). Single plants from the parental lines were included as controls. Plants were arranged in a completely randomized design. BC_3_F_2_ generation consisted of segregating individual plants; therefore, evaluation was used primarily for marker-assisted and phenotypic selection rather than formal replicated comparison among fixed genotypes.

Each BC_3_F_2_ plant was evaluated for marker genotype at the targeted FHB resistance loci (*Fhb7*, *Fhb1*, *Qfhb.rwg-5A.1*, and *Qfhb.rwg-5A.2)*, Type II FHB disease severity (DS), and visual plant phenotype. Leaf tissue was collected at the two-leaf stage for marker analysis. Visual phenotype was scored on a 1–5 scale, where 1 represented poor agrotype and 5 represented the most desirable agrotype. Phenotypic assessment considered overall plant vigor, plant architecture, spike development, and suitability for continued selection. Marker data, DS, and phenotypic scores were used jointly to select 32 BC_3_F_2_:_3_ families for further evaluation.

### Greenhouse evaluation of selected BC_3_F_2:3_ families

The 32 selected BC_3_F_2_:_3_ families were divided into two groups for further evaluation. The first group consisted of 22 families that were promising sources of additional marker combinations involving two or more of *Fhb1*, *Fhb7*, *Qfhb.rwg-5A.1*, and *Qfhb.rwg-5A.2*, but were not fixed at all target loci. A total of 362 individual plants from these 22 families were grown in the greenhouse, re-tested for marker genotype, and assessed for agronomic traits at or beyond the late dough stage.

Traits recorded for these plants included the number of spikes per plant, days from planting to heading, plant height, spikelets per spike, seed set percentage, and 100-kernel weight. Plant height was measured in centimeters from the base of the plant to the tip of the primary spike, excluding awns. Spikelets per spike were counted on the main tiller. Seed set percentage was calculated as the proportion of florets on the main tiller that produced seed. The 100-kernel weight was determined by weighing and counting seeds from each plant. An agrotype index was calculated using the following formula:

Agrotype index = (number of spikes × spikelets per spike × seed set percentage × 100-kernel weight)/100.

Marker and agronomic data were used jointly to select 94 BC_3_F_2_:_4_ families carrying different combinations of FHB resistance loci (**Supplementary Table 1**).

The second group consisted of 10 BC_3_F_2_:_3_ families that were already homozygous for one or more target marker loci involving *Fhb7*, *Qfhb.rwg-5A.1*, and/or *Qfhb.rwg-5A.2*. These families were re-evaluated for Type II FHB resistance in a replicated greenhouse trial. Five pots were used as replicates for each entry, with five to six seeds planted per pot. Entries were arranged in a completely randomized design.

### FHB inoculation and disease assessment

Type II FHB resistance was evaluated using a single-floret inoculation method (Stack, 1989). At anthesis, three to five spikes per plant were tagged and inoculated. A central spikelet of each spike was injected with approximately 10 µL of an *F. graminearum* conidial suspension using a syringe. Care was taken to minimize physical injury to the spikelet during inoculation.

The inoculum consisted of a mixture of four *F. graminearum* isolates, *Fg*08_13, *Fg*10_135_5, *Fg*10_124_1, and *Fg*13_79, originally collected from symptomatic wheat spikes in a farmer’s field across North Dakota and maintained in the culture collection by Dr. Shaobin Zhong (Puri and Zhong, 2010), Department of Plant Pathology, North Dakota State University. The conidial suspension was prepared at approximately 100,000 conidia mL^−1^. Immediately after inoculation, spikes were covered with moist plastic bags for 48–72 h to maintain high humidity and promote infection. Greenhouse temperature during the post-inoculation period was maintained at approximately 22–25 °C.

Disease symptoms were evaluated 21 days after inoculation. DS was calculated as the proportion of bleached spikelets per inoculated spike, following Rawat et al. (2016). For each inoculated spike, DS was calculated as:


DS=(number of bleached spikelets/total number of spikelets)×100.

### Statistical analysis

Statistical analyses were performed using R version 4.3.3 in RStudio and SAS version 9.4 M5 (https://documentation.sas.com/doc/en/pgmsascdc/9.4_3.5/whatsdiff/n1e6izkiq9zivtn164bwbvo6okhf.htm). Segregation of marker alleles was evaluated using chi-square goodness-of-fit tests. Expected Mendelian ratios were tested according to the segregation pattern of each marker and generation. Codominant marker data in segregating BC_3_F_2_ families were tested against the expected 1:2:1 ratio, while dominant marker segregation in backcross-derived generations was evaluated according to the expected backcross segregation ratio where appropriate.

For analysis of marker effects on Type II FHB DS, marker genotypes were numerically coded as 0, 1, or 2, corresponding to homozygous susceptible, heterozygous, and homozygous resistant classes, respectively, when codominant marker data were available. Pearson’s correlation coefficients were calculated to assess the relationship between marker genotype and DS for each target locus. Beta regression was used to model the relationship between DS and multiple marker loci, with DS expressed as a proportional response variable. Beta regression analyses were performed using the betareg package in R (https://cran.r-project.org/web/packages/betareg/index.html). Although DS data were not normally distributed, analysis of variance (ANOVA) was performed for the replicated greenhouse evaluations because the *F*-test is generally considered robust to moderate departures from normality when sample sizes are adequate and variance assumptions are reasonably satisfied (Blanca et al., 2017). Each of the inoculated spikes was considered a single experimental unit. *Post-hoc* mean comparisons were conducted using Bonferroni adjustment at the 95% confidence level. Chi-square tests for marker segregation were conducted in Microsoft Excel.

## Results

### Marker analyses to detect *Fhb7* in the backcross F_1_ generations

In the BC_1_F_1_, *Fhb7* positive and negative parental controls and 60 BC_1_F_1_ plants (**Figure 1**) were analyzed using the dominant *Fhb7* STS marker WGC2315. The WGC2315 amplicon in positive controls was 941 bp, as reported by Zhang et al. (2022). Among the BC_1_F_1_ progenies, 30 plants were positive for *Fhb7* and 30 were negative, consistent with monogenic inheritance. Analysis of the BC_2_F_1_ and BC_3_F_1_ generations with marker WGC2315 revealed that 11 of the 35 BC_2_F_1_ plants and 18 of the 51 BC_3_F_1_ plants tested positive for the *Fhb7* diagnostic marker. All of the resistant backcross F_1_ were grown, but only those with the best agrotypes were used for ongoing crosses/selection. Nine BC_3_F_1_ plants, heterozygous for *Fhb7*, were kept and allowed to self-pollinate (**Table 1**). The complete BC_3_F_2_ data (marker, FHB resistance, and phenotypic) that were gathered are provided in **Supplementary Table 2**.

### Marker analyses to detect *Fhb7*, *Qfhb.rwg-5A.1*, *Qfhb.rwg-5A.2*, and *Fhb1* in the BC_3_F_2_

In the BC_3_F_2_ generation, a new *Fhb7* STS marker set that allows for co-dominant detection (Cai et al., 2024) was used to test for *Fhb7*. A total of 322 BC_3_F_2_ plants and 10 parents used in the modified backcross scheme (**Figure 1**) were analyzed using the new marker. The parents included nine winter wheats lacking *Fhb7* and the spring wheat *Fhb7* donor parent, WGC002. Each winter wheat parent produced a single 135-bp band, whereas WGC002 displayed a single 294-bp band. The marker banding patterns matched those reported by Cai et al. (2024). Genotyping of the 322 BC_3_F_2_ plants revealed that 73 were likely *Fhb7* homozygotes, as indicated by the presence of the 294-bp band. A total of 85 plants exhibited the 135-bp band, indicating the absence of *Fhb7* and thus recessive homozygosity. The remaining 164 plants were heterozygotes, displaying both bands. A chi-square goodness-of-fit test confirmed a 1:2:1 Mendelian segregation ratio at the 95% confidence level (χ^2^ = 0.99, *p* > 0.05).

*Qfhb.rwg-5A.1* and *Qfhb.rwg-5A.2* QTL segregated in four of the six crosses shown in **Table 1**. Crosses 23K466 and 23K478 lacked these QTL, and their F_2_ progeny showed only the recessive polymorphisms associated with both loci. Marker *Xbarc186* was used to detect *Qfhb.rwg-5A.1* and segregated in the 222 BC_3_F_2_ plants. Duplicate assays at the USDA-ARS Biosciences Research Lab in Fargo and Winter Wheat Laboratory gave the same results. Based on *Xbarc186* polymorphisms, 56 BC_3_F_2_ plants were homozygous resistant, 69 were homozygous susceptible, and 97 were heterozygous, conforming to a 1:2:1 Mendelian ratio (χ^2^ = 5.05, *p* > 0.05). For *Qfhb.rwg-5A.2*, marker Gpw2136 detected the QTL in three parents known to possess it and was polymorphic in 218 BC_3_F_2_ plants from the four crosses. A total of 52 plants were homozygous resistant, 100 were heterozygous, and 66 were homozygous susceptible, also segregating as expected (χ^2^ = 3.28, *p* > 0.05).

The *Fhb1* gene segregated exclusively within cross 23K487. Genotyping of these 49 individuals using the markers *Fhb1-TaHRC* and *IFA-FM227* identified 15 homozygous resistant, 24 heterozygous, and 10 homozygous susceptible individuals. The observed segregation pattern did not deviate significantly from the expected 1:2:1 ratio (χ^2^ = 1.04, *p* > 0.05).

### BC_3_F_2_: Evaluation for FHB resistance and plant phenotype

Of the 322 BC_3_F_2_ plants and 10 parental controls that were grown for Type II FHB resistance testing, only 145 BC_3_F_2_ plants and 6 parents were fully vernalized. This was the result of cold-room malfunction combined with a strong vernalization requirement in some genotypes, particularly crosses 23K466 and 23K478 (**Table 1**). Plants that could not be properly evaluated due to their late flowering were not tested. To assess whether this equipment failure introduced systematic selection bias, chi-square tests of homogeneity were performed comparing marker genotype frequencies between the fully vernalized subset (*n* = 145) and the non-vernalized subset (*n* = 177) for all four marker loci. Three of four markers showed no significant difference between subsets: *Fhb7* (χ^2^ = 5.2, df = 2, *p* = 0.074), *Qfhb.rwg-5A.2* (χ^2^ = 1.25, df = 2, *p* = 0.534), and *Fhb1* (χ^2^ = 0.85, df = 2, *p* = 0.654) (**Supplementary Table 3**). A borderline significant difference was detected for *Qfhb.rwg-5A.1* (χ^2^ = 6.62, df = 2, *p* = 0.037), driven primarily by underrepresentation of the AA homozygous class in the vernalized subset (18.8% vs. 33.7%). Given that four simultaneous tests were performed, this single borderline result may reflect Type I error. Within the vernalized subset, goodness-of-fit tests (**Supplementary Table 4**) confirmed that all four markers conformed to the expected 1:2:1 Mendelian segregation ratios (*Fhb7*: χ^2^ = 3.99, *p* = 0.14; *Qfhb.rwg-5A.1*: χ^2^ = 3.86, *p* = 0.14; *Qfhb.rwg-5A.2:* χ^2^ = 2.43, *p* = 0.30; *Fhb1*: χ^2^ = 0.21, *p* = 0.90), confirming representative Mendelian segregation at all loci in the evaluated subset.

**Figure 2:**
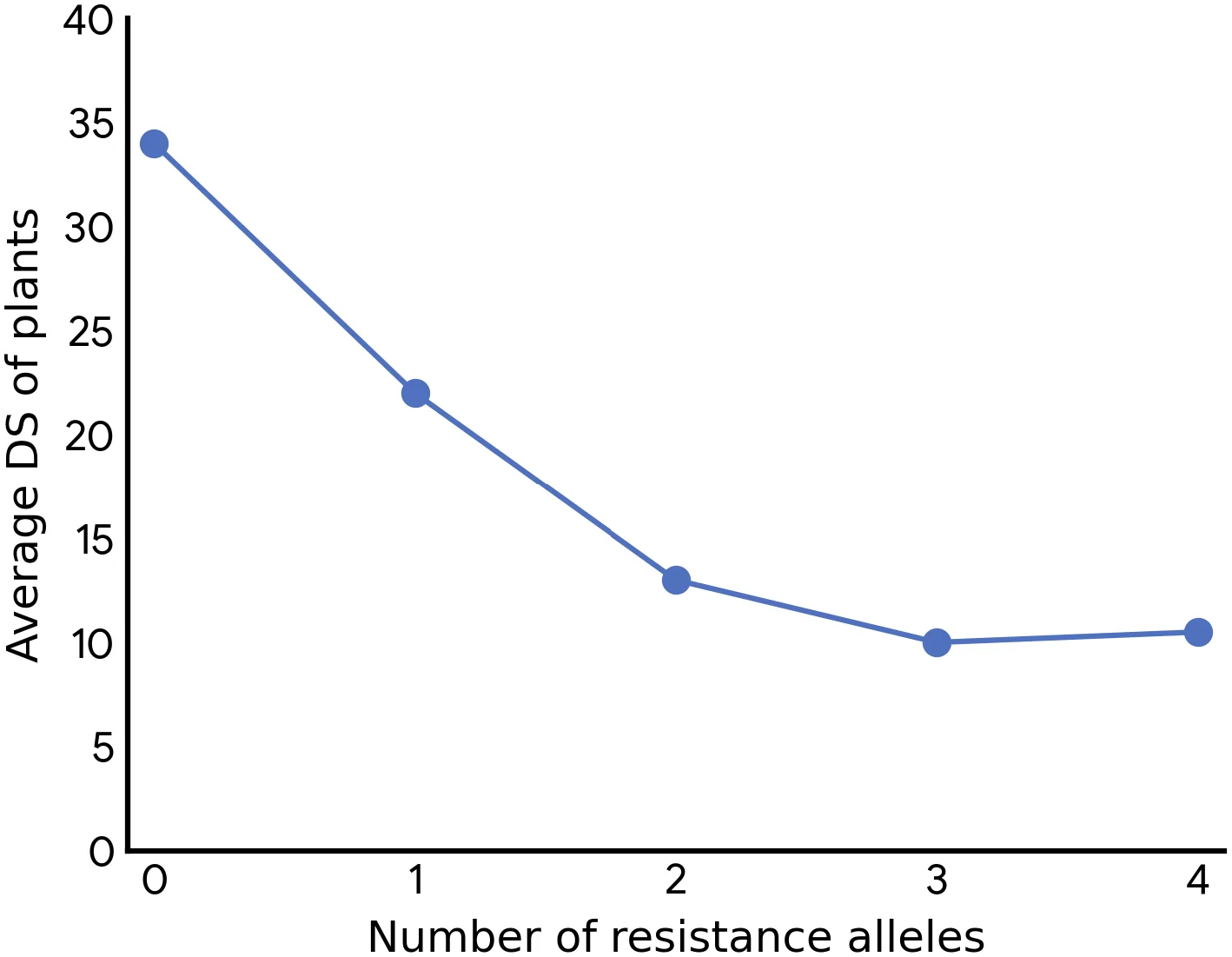
Mean FHB disease severity of 133 BC3F2 that were grouped (with the use of marker data) into five classes of plants based on the likely presence of resistance alleles at both the Fhb7 and Qfhb.rwg-5A.2 loci.

**Figure 3:**
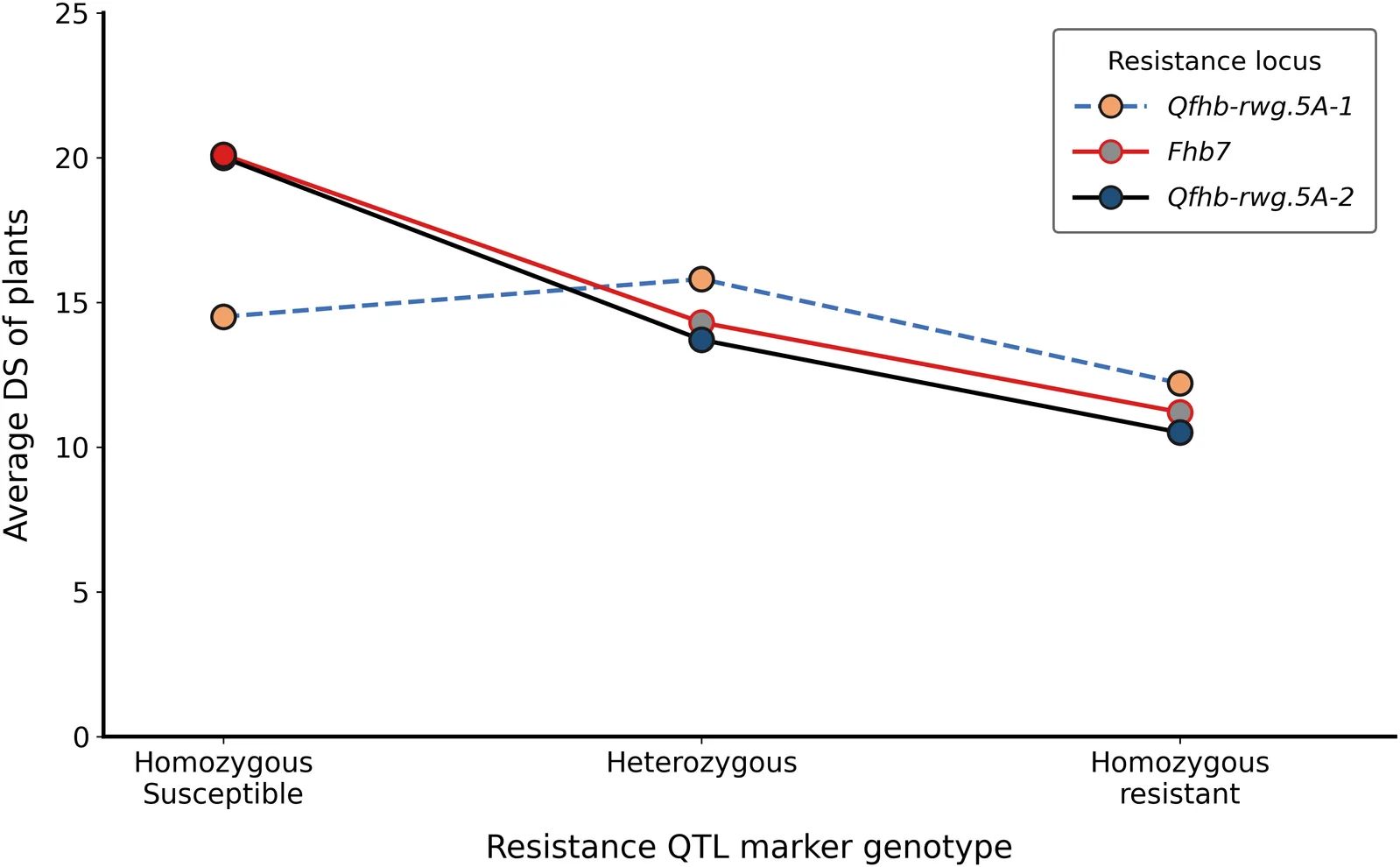
Mean FHB disease severity of 133 BC_3_F*_2_* that were grouped into likely homozygous susceptible, heterozygous, and homozygous resistant plants with the use of marker data for *Fhb7*, *Qfhb.rwg-5A.1*, and *Qfhb.rwg-5A.2*.

DS ranged from 4.6% to 94.4% (mean = 15.5%). A total of 73 plants had DS below 10%, and 5 plants had DS below 5%. Most of the latter 78 individuals had at least one allele of *Fhb7*, while 11 lacked *Fhb7* but had either one or both of *Qfhb.rwg-5A.1* and *Qfhb.rwg-5A.2*. Within crosses 23K468, 23K461, 23K472, and 23K487 (**Table 1**), 133 plants could be scored for FHB resistance and produced an average DS of 14.79%. Resistance QTL *Fhb7*, *Qfhb.rwg-5A.1*, and *Qfhb.rwg-5A.2* segregated in each of the four crosses, and their relative effects could be compared across all 133 plants. In addition, it was highly likely that uncharacterized background FHB resistance QTL derived from ND Noreen segregated in each of the four crosses. With respect to this set of plants, the marker results were used to predict the number of dominant alleles (susceptible homozygote = 0, heterozygote = 1, and resistant homozygote = 2) contributed by resistance QTL *Fhb7*, *Qfhb.rwg-5A.1*, and *Qfhb.rwg-5A.2* in each of the 133 BC_3_F_2_ plants. This total number of resistance alleles in a plant varied from 0 to 4 in the data set and was plotted against the average DS (**Figure 2**). A gradual decrease in average DS was observed as the number of resistance alleles increased. It is very likely that undetected background resistance QTL also contributed to the total resistance, complementing and strengthening the expression of *Fhb7*, *Qfhb.rwg-5A.1*, and *Qfhb.rwg-5A.2* (Ganaparthi et al., 2023). With regard to the individual contributions to Type II FHB resistance (**Figure 3**), both *Fhb7* and *Qfhb.rwg-5A.2* significantly reduced DS. For *Fhb7*, mean DS declined from 20.3% (*rr*) to 10.3% (*RR*) (*r* = –0.25, *p* < 0.001); for *Qfhb.rwg-5A.2*, it declined from 20.4% to 11.4% (*r* = –0.27, *p* < 0.001). *Qfhb.rwg-5A.1* appeared to contribute the least to Type II resistance. The average DS values were 14.5% for susceptible homozygotes, 15.9% for heterozygotes, and 12.2% for resistant homozygotes, indicating a relatively small effect of this marker on DS (*r* = 0.07, *p* = 0.38). This QTL, suspected to be the same as *Qfhs.ifa-5A* (Chu et al., 2011), has been reported to confer stronger Type I rather than Type II resistance (Buerstmayr et al., 2009).

A beta regression analysis was conducted using the betareg package in R (Cribari-Neto and Zeileis, 2010), with DS as the proportional response variable (logit link for the mean and identity link for the precision parameter). The analysis was restricted to the 133 plants from crosses 23K468, 23K461, 23K472, and 23K487, in which all three QTL segregated simultaneously. This excluded families 23K466 (*n* = 1) and 23K478 (*n* = 11), reducing the 145 fully vernalized, FHB-phenotyped individuals to the 133 used in the beta regression. Marker genotypes were coded additively (AA = 2, Aa = 1, aa = 0), reflecting an additive genetic model. A full four-locus model including *Fhb1* was first evaluated; a likelihood ratio test confirmed that *Fhb1* did not significantly improve model fit (χ^2^ = 0.32, df = 1, *p* = 0.572), with AIC favoring the three-locus model (−237.3 vs. −235.7). The final model (DS ~ *Fhb7* + *Qfhb.rwg-5A.1* + *Qfhb.rwg-5A.2*) converged after 12 BFGS iterations and 3 Fisher scoring iterations. Two predictors were statistically significant: *Fhb7* [β = −0.314, SE = 0.109, *z* = −2.89, *p* = 0.004, 95% CI (−0.527, −0.101)] and *Qfhb.rwg-5A.2* [β = −0.292, SE = 0.096, *z* = −3.03, *p* = 0.002, 95% CI (−0.481, −0.103)]. *Qfhb.rwg-5A.1* was not significant [β = −0.088, SE = 0.100, *z* = −0.89, *p* = 0.374, 95% CI (−0.283, +0.107)]. The precision parameter was φ = 8.37 [SE = 1.029, 95% CI (6.35, 10.39)], indicating a well-concentrated beta distribution without overdispersion. The model pseudo-*R*^2^ was 0.122, indicating that the three marker loci explained 12.2% of phenotypic variation in DS.

Since *Fhb1* segregated in cross 23K487 only, all four QTL could be compared with one another in the 21 fully vernalized genotypes of this cross only. Although *Fhb1* showed a moderate negative correlation with DS (*r* = –0.32), the relationship was not statistically significant (*p* = 0.16), likely because of the small sample size (21 plants). No meaningful comparisons were possible with regard to the remaining QTL either.

Visually assessed phenotypic scores (1 to 5) were assigned to 270 of the 322 plants. There were 31 plants with phenotypic scores of 4 or better. A total of 44 plants had scores of 2.5 to 3.5, whereas the remaining 83 plants had scores of 2 or lower. Thirty-two BC_3_F_2:3_ families were selected for further testing: (i) Twenty-two families were heterozygotes for useful FHB resistance QTL, had phenotypic scores of 4–4.5, and/or had comparatively low DS (5.0%–17.3%). These 22 families derived from crosses 23K461 (10), 23K472 (5), 23K478 (4), and 23K487 (3) were used for doing another round of marker selection and phenotypic evaluation. (ii) Ten BC_3_F_2:3_ families were homozygous at the marker loci of one or more of *Fhb7*, *Qfhb.rwg-5A.1*, and *Qfhb.rwg-5A.2*. In addition, they had good phenotypic scores (3.5–4.5) and/or low DS (6.02%–12.7%). The 10 families were derived from crosses 23K461 (4), 23K466 (2), and 23K478 (4) and were retested in a replicated FHB resistance trial in an attempt to confirm their strong resistance.

### Evaluation of 22 BC_3_F_2:3_ families for FHB resistance markers and plant phenotype

On average, 17 plants per family were grown in a greenhouse, resulting in 362 individual plants and parental controls. Marker analyses were performed at the USDA-ARS Biosciences Research Lab in Fargo, and plant agronomic measurements were recorded. Marker data of both the BC_3_F_2_ and BC_3_F_2:3_ generations were compared across the 22 families to confirm classification into likely resistant homozygotes and heterozygotes. A total of 94 plants were selected on the following criteria: First, the presence of *Fhb7*, as its introgression was the primary goal of the study. The selected group consisted of 90 *Fhb7* homozygotes and four *Fhb7* heterozygotes (**Supplementary Table 1**). Second, the presence of one or more of *Fhb1*, *Qfhb.rwg-5A.1*, or *Qfhb.rwg-5A.2*, either in the homozygous or heterozygous state. For *Qfhb.rwg-5A.1*, 45 plants were homozygous for the resistance allele, 28 were heterozygous, and 21 lacked the marker. For *Qfhb.rwg-5A.2*, 4 tested as homozygous resistant, 69 plants were heterozygous, and 21 lacked the allele. With respect to *Fhb1*, 38 plants were tested as homozygous resistant, 18 were heterozygous, and 38 were homozygous susceptible. Third, favorable plant height and agrotype score were considered. Plants taller than 90 cm were rejected to avoid lodging problems when taken to the field. None of the 48 plants from cross 23K478 were retained due to being too tall or having poor agrotypes. Agrotype index scores ranged from 71 to 1,588 (mean = 323), with reference scores of 376 for 19Nord-124, 346 for Winner, and 300 for ND Noreen. The average agrotype index for the selected 94 BC_3_F_2:3_ plants was 267. The 94 selections were derived from three of the four crosses: 23K461, 23K472, and 23K487.

### Retesting of 10 BC_3_F_2:3_ families to confirm their FHB Type II resistance

**Figure 4:**
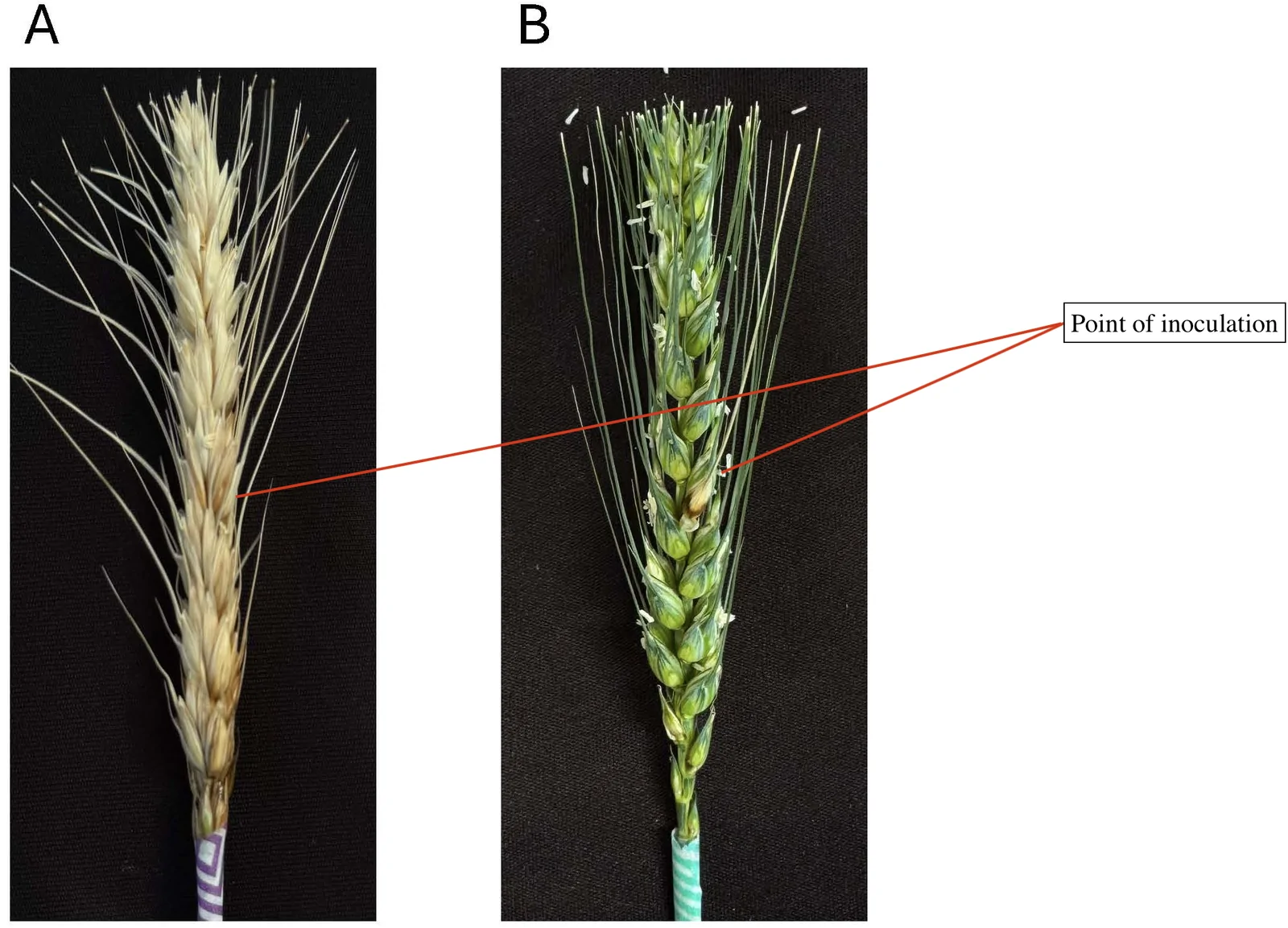
Wheat spikes showing different FHB resistance: **(A)** SY monument (highly susceptible; average DS = 73.06%); **(B)** entry 3 (highly resistant; average DS = 5.62%).

The marker analyses suggested that 9 of the 10 BC_3_F_2:3_ families were homozygous for *Fhb7* (**Table 2**). One of the nine lines was also homozygous for *Qfhb.rwg-5A.1* and another was also homozygous for *Qfhb.rwg-5A.2.* The remaining line lacked *Fhb7* but was homozygous for both *Qfhb.rwg-5A.1* and *Qfhb.rwg-5A.2*. Eleven controls (**Table 2**) included Chinese Spring and WGC002 as well as nine HWW cultivars currently grown in ND. Average DS values of the 21 trial entries ranged from 5.62% to 73.06%. ANOVA of the DS data confirmed highly significant (*p* < 0.0001) genetic differences in FHB Type II resistance among the 21 entries (**Table 3**). Examples of extreme FHB disease symptoms in a highly susceptible spike of a control cultivar and a highly resistant spike of a *Fhb7* homozygote are shown in **Figure 4**. Among the cultivated controls, ND Noreen (18.10%) and Winner (24.50%) were the most resistant, while SY Monument (73.06%) was the most susceptible. Jerry and Emerson showed intermediate resistance.

**Table 2:** Type II Fusarium head blight disease severity and agronomic phenotype of 10 selected BC_3_F_2_:_3_ families and 11 control entries evaluated in a replicated greenhouse trial.

Entry no.	Pedigree/cultivar	Homozygous for resistance QTL markers			DS^2^ range (%)	Height (cm)	Phenotypic score	Mean DS ± SE^3^
1	WGC002/ND Allison/2*Winner/3/20M1-58-27	_	*5A.1^1^*	*5A.2^1^*	4.55–55.56	88.00	5	13.12 ± 2.19^efg^
2	WGC002/ND Allison/2*Winner/3/20M1-58-27	*Fhb7*	*5A.1^1^*	_	4.55–20.00	104.00	4	6.57 ± 0.28^g^
3	WGC002/ND Allison/2*Winner/3/20M1-58-27	*Fhb7*	_	*5A.2^1^*	4.55–8.33	94.00	3	5.62 ± 0.11^g^
4	WGC002/ND Allison/2*Winner/3/ND Allison	*Fhb7*	_	_	5.00–33.33	82.00	4	8.96 ± 0.84f^g^
5	WGC002/ND Allison/2*Winner/3/ND Allison	*Fhb7*	_	_	5.00–50.00	84.50	3	12.00 ± 1.12^efg^
6	WGC002/ND Allison//Winner/3/ND Noreen/4/Midland	*Fhb7*	_	_	4.55–21.43	105.00	3.5	7.00 ± 0.39^g^
7	WGC002/ND Allison//Winner/3/ND Noreen/4/Midland	*Fhb7*	_	_	4.55–50.00	112.00	3	9.58 ± 1.11^fg^
8	WGC002/ND Allison/2*Winner/3/20M1-58-27	*Fhb7*	_	_	4.55–35.71	94.00	4	7.65 ± 0.65^g^
9	WGC002/ND Allison//Winner/3/ND Noreen/4/Midland	*Fhb7*	_	_	4.55–16.67	105.00	3	6.12 ± 0.19^g^
10	WGC002/ND Allison//Winner/3/ND Noreen/4/Midland	*Fhb7*	_	_	4.55–41.67	89.00	3	8.65 ± 1.03^fg^
	Chinese Spring	_	_	_	5.56–100.00	105.00	0	61.33 ± 4.75^bcd^
	ND Allison	_	_	_	5.00–100.00	88.00	3	35.49 ± 3.34^d^
	Winner	_	_	_	5.00–100.00	78.00	3	24.50 ± 3.24^e^
	ND Noreen	_	_	_	5.00–100.00	89.00	2	18.10 ± 2.32^ef^
	Midland	_	_	_	5.00–100.00	83.50	2	54.65 ± 4.75^abc^
	WGC002	*Fhb7*	_	_	5.56–10.00	100.00	0	7.35 ± 0.13^g^
	Jerry	_	_	_	5.00–100.00	95.00	2	43.10 ± 4.00^bcd^
	SY Monument	_	_	_	5.56–100.00	58.00	4	73.06 ± 4.19^a^
	Keldin	_	_	_	4.17–100.00	75.50	3	29.51 ± 2.50^d^
	Emerson	_	_	_	5.56–100.00	86.00	3	38.39 ± 3.95^cd^
	Andes	_	_	_	5.00–100.00	76.00	3	57.12 ± 4.19^ab^

^1^Qfhb.rwg-5A.1 = 5A.1, Qfhb.rwg-5A.2 = 5A.2.

^2^DS, disease severity (%).

^3^Means followed by the same letter were not significantly different at the 99.9% confidence level, based on the Bonferroni test for mean separation.

**Table 3:** Analysis of variance for type II Fusarium head blight disease severity among 21 entries evaluated in a replicated greenhouse trial using a completely randomized design.

Source	df	Type III SS	Mean square	*F* value	*p*-value
Entry	20	571260	28,563	64.64	<0.0001***
Error	1295	572224	442		

***Indicates statistical significance at the 99.9% confidence level (corresponds to *p* < 0.001).

The average DS of Chinese Spring was 61.33% as compared to that of WGC002, which was 7.35% (**Table 2**). The average DS of the nine *Fhb7*-positive BC_3_F_2:3_ lines ranged from 5.62% to 12.0%. The average DS of nine cultivars currently grown in ND and lacking *Fhb7* ranged from 18.10% to 73.06%, suggesting a strong contribution of *Fhb7* to Type II resistance of families with the gene. Line 3 had the lowest mean DS (5.62%) and possesses both *Fhb7* and *Qfhb.rwg-5A.2*.

The phenotypic scores of the 10 B_3_F_2:3_ families ranged from 3 to 5, which were very comparable to those of the cultivated controls (range = 2–4). The average heights of the 10 families ranged from 82 to 112 cm. This suggests that the poor plant architecture associated with the *Fhb7* donor germplasm (Zhang et al., 2022) had largely been removed during backcrossing. F_4_ populations derived from top-performing plants of each of the 10 families will be employed for pure line selection and broader field evaluation, including assessments of winter hardiness, general disease resistance, yield, and end-use quality.

## Discussion

*Fhb7* is a valuable addition to the repertoire of FHB resistance genes available to wheat breeders, but its donor line, WGC002, carries an undesirable CS derived background. This gene confers strong Type II resistance through diverse mechanisms. Its defining biochemical function is GST-mediated detoxification of the trichothecene mycotoxin (Wang et al., 2020), which operates alongside antioxidant and cell-wall responses as part of a broader defense network (Fanelli et al., 2023). Although DON was not measured here, the trichothecene-detoxifying activity of *Fhb7* (Wang et al., 2020) would be expected to lower mycotoxin contamination of the grain and to limit fungal spread within the spike. This pre-breeding project aimed to rapidly establish *Fhb7* throughout NDSU HWW breeding material while simultaneously pyramiding it with other effective resistance loci.

Backcrossing is the simplest way to achieve this and at the same time get rid of deleterious genes derived from the WGC002 donor background. Introgression through regular backcrosses can severely narrow genetic variability within the breeding pool; however, modified backcrosses (Fehr, 1991) result in a broad spectrum of genetically diverse pre-bred lines that better conserve existing genetic variability. Carefully chosen, diverse, and well-adapted backcross parents furthermore allowed for pyramiding *Fhb7* with other effective FHB resistance QTL and even un-characterized background resistance genes. This study recovered 94 genetically diverse BC_3_F_2_:_3_ selections combining *Fhb7* with one or more of *Fhb1*, *Qfhb.rwg-5A.1*, and *Qfhb.rwg-5A.2*, including a subset in which three resistance loci were stacked. These pyramids may be useful because they bring together loci associated with different aspects of FHB resistance, including toxin detoxification by *Fhb7* and reduced disease spread associated with *Fhb1* and the 5A loci (Buerstmayr et al., 2009). This pool of pre-bred lines will serve to yield an advanced set of *Fhb7* parents that can be used to incorporate the resistance into a still wider portion of HWW germplasm.

Greenhouse Type II resistance evaluation revealed that more than 75% of the BC_3_F_2_ plants carrying *Fhb7* exhibited disease severities below 15%, consistent with the expected level of Type II resistance and comparable to previously reported values (Wang et al., 2020; Cai et al., 2024). Based on an average spikelet number of ~16.7 per spike, infection of a single spikelet corresponded to roughly 6% DS, which provided a rough estimate of the lower limit of the rating scale. The resistance levels in the nine BC_3_F_2:3_ populations of **Table 2** did not differ significantly and clustered at the lower end of the disease rating scale; thus, it was not possible to draw conclusions regarding the presence and extent of gene complementation to achieve higher levels of resistance.

In contrast, the segregating BC3F2 populations allowed dosage effects to be examined directly. Both *Fhb7* and *Qfhb.rwg-5A.2* showed a progressive, allele-dosage-dependent reduction in DS (rr → Rr → RR), consistent with additive gene action. When considered together, DS declined from 32.20% in plants lacking resistance alleles at both loci to 10.53% in double-resistant homozygotes, supporting an additive contribution of these loci to Type II resistance.

These findings are consistent with previous studies showing that FHB resistance in wheat is quantitatively inherited and that pyramiding multiple resistance loci generally produces stronger and more stable resistance than relying on a single major QTL. For example, pyramid combinations of *Fhb1*, *Fhb4*, and *Fhb5* have been shown to improve FHB resistance by 95% in modern wheat backgrounds (Zhang et al., 2021), while stacking *Fhb1*, *Fhb2*, and *Fhb5* provided superior multi-environment field resistance compared with single- or dual-gene introgressions (Dai et al., 2022).

*Fhb7* resistance in our material derives from WGC002 (Cai et al., 2024), which carries the *Th. elongatum*-derived allele *Fhb7^The2^* on a recombined 7B segment. This same allele was recently shown to confer higher Type II resistance when combined with *Fhb1* than either gene alone, with combined lines at 8%–13% for infected spikelets versus 23–36% for *Fhb1*-only and 29% for *Fhb7^The2^*-only lines (Dvorak et al., 2025). Notably, an earlier attempt to combine *Fhb1* with the *Th. ponticum* allele *Fhb7^Thp^* did not exceed *Fhb1* alone (Guo et al., 2015), indicating that this benefit is allele-specific and that the *Fhb7^The2^* allele in our material is the favorable one.

Pyramiding gains can nonetheless vary with genetic background, evaluation method, and environment (Buerstmayr et al., 2009). Brar et al. (2019) illustrated the influence of genetic background: when *Fhb1*, *Fhb2*, and *Fhb5* were introgressed into two near-isogenic line (NIL) populations, the resulting resistance differed markedly between backgrounds. In the susceptible cultivar CDC Alsask, both single genes and their combinations significantly reduced disease, whereas in the moderately susceptible CDC Go, only the full three-gene stack improved resistance. The importance of background is also evident within our own program: the ND Noreen background was previously found to complement the PI 277012-derived resistance underlying *Qfhb.rwg-5A.1* and *Qfhb.rwg-5A.2* (Ganaparthi et al., 2023). Taken together, the present study suggests that *Fhb7* is a strong contributor to Type II resistance, but that its value will likely be greatest when combined with complementary loci (e.g., *Qfhb.rwg-5A.2* and *Fhb1*) and favorable background resistance from adapted winter wheat parents.

The beta regression model explained 12.2% of the phenotypic variation in DS (pseudo-*R*^2^ = 0.122), with the significant precision parameter (φ = 8.37) indicating an appropriate model fit. The low *R*^2^ reflects the quantitative nature of FHB resistance rather than a methodological shortcoming. Several of the most resistant families are thought to carry uncharacterized background resistance derived from ND Noreen on the basis of their pedigree, although this was not experimentally confirmed; because such background QTL were not genotyped, they could not be captured by the model and represent a plausible but untested contributor to the unexplained variance. Additional variance is attributable to the inherent variability of single-spike greenhouse inoculations. The low model *R*^2^ underscores the need for multi-environment field evaluation and larger populations to fully characterize the contributions of individual and pyramided resistance loci.

Agronomic acceptability of selected plants was a major consideration throughout the backcrossing process. Early backcross progenies showed substantial variability in plant type, plant height, straw strength, and spike characteristics. Rigorous phenotypic selection was done in the BCF_1_ generations and subsequent evaluations of the later generations. No obvious adverse phenotypic effects were associated with the *Fhb7* translocation in either of the BC_3_F_2_ and BC_3_F_2:3_ generations, consistent with the observations of Ceoloni et al. (2017) and Wang et al. (2020).

Overall, this research provided a strong foundation for the introgression of *Fhb7* into the NDSU winter wheat breeding program. The 104 genotypes identified in this study represent promising pre-breeding material carrying pyramids of FHB resistance loci in adapted HWW genetic backgrounds. Because all evaluations were conducted exclusively under controlled greenhouse conditions using Type II resistance assays, these selections should be considered candidates for further evaluation rather than validated elite resistance sources. FHB resistance is highly environment-dependent, and greenhouse performance does not consistently predict field epidemic response, Type I resistance, or DON accumulation. Multi-environment field trials and DON quantification are the necessary next steps before these lines can be recommended for direct use in crossing programs. Future work will focus on developing pure lines from these selections, assessing their performance across diverse field environments, and using them in new crosses. Ultimately, this pre-breeding effort supports the long-term goal of developing winter wheat cultivars with durable, broad-spectrum FHB resistance, particularly regions prone to FHB epidemics.

## Data Availability

The original contributions presented in the study are included in the article/**Supplementary Material**. Further inquiries can be directed to the corresponding author.
