# Supplementary material for: Pre-breeding to establish Fhb7 in winter wheat using marker-assisted, modified backcrossing
Source: Front Plant Sci. 2026 Aug 26;17:1863042. doi: 10.3389/fpls.2026.1863042 (PMC13553846; doi:10.3389/fpls.2026.1863042)
Supplement: Supplementary file 1 [file Table1.docx]

**Supplementary Table 1.** Summary of marker data, FHB resistance, and phenotypic traits for 322 BC₃F₂ plants and parental controls. FHB resistance genes are coded as 0 (no resistance allele), 1 (one resistance allele present), and 2 (two resistance alleles present).The corresponding pedigrees are given in Table 1.

| **Plant No** | **Entry** | ***Fhb7*** | ***5a.1^1^*** | ***5a.2^2^*** | ***Fhb1*** | **Av. DS^3^** | **Pheno^4^** |
| --- | --- | --- | --- | --- | --- | --- | --- |
| **524** | Chinese Spring | 0 | 0 | 0 | 0 | 83.87 | _ |
| **525** | ND Allison | 0 | 0 | 0 | 0 | _ | _ |
| **526** | Winner | 0 | 0 | 0 | 0 | _ | _ |
| **527** | ND Noreen | 0 | 0 | 0 | 0 | 33.33 | _ |
| **528** | 19Nord-124 | 0 | 2 | 0 | 2 | _ | _ |
| **529** | 20M1-23-6 | 0 | 2 | 2 | 0 | 19.95 | _ |
| **530** | 20M1-58-10 | 0 | 2 | 2 | 0 | 19.97 | _ |
| **531** | 20M1-58-27 | 0 | 2 | 2 | 0 | 14.81 | _ |
| **532** | Midland | 0 | 0 | 0 | 0 | _ | _ |
| **533** | WGC002 | 2 | 0 | 0 | 0 | 15.29 | _ |
| **534** | 23K468-10-1 | 0 | 1 | 1 | 0 | _ | _ |
| **535** | 23K468-10-2 | 1 | 0 | 0 | 0 | 6.35 | _ |
| **536** | 23K468-10-3 | 1 | 1 | 0 | 0 | _ | _ |
| **537** | 23K468-10-4 | 1 | 1 | 1 | 0 | 5.29 | _ |
| **538** | 23K468-10-5 | 2 | 1 | 0 | 0 | 5.96 | 1 |
| **539** | 23K468-10-6 | 2 | 1 | 1 | 0 | 18.06 | 3 |
| **540** | 23K468-10-7 | 1 | 2 | 1 | 0 | 24.42 | 2 |
| **541** | 23K468-10-8 | 1 | 0 | 0 | 0 | 31.82 | _ |
| **542** | 23K468-10-9 | 0 | 2 | 2 | 0 | 14.13 | 1.5 |
| **543** | 23K468-10-10 | 2 | 1 | 2 | 0 | 23.59 | 3 |
| **544** | 23K468-10-11 | 2 | 0 | 2 | 0 | 6.52 | 2 |
| **545** | 23K468-10-12 | 1 | 1 | 1 | 0 | 6.52 | _ |
| **546** | 23K468-10-13 | 1 | 1 | 1 | 0 | 11.47 | _ |
| **547** | 23K468-10-14 | 1 | 0 | 1 | 0 | 10.73 | _ |
| **548** | 23K468-10-15 | 1 | 1 | 2 | 0 | 7.94 | 3 |
| **549** | 23K468-10-16 | 1 | 2 | 1 | 0 | 7.1 | 2.5 |
| **550** | 23K468-10-17 | 1 | 1 | 1 | 0 | 8 | _ |
| **551** | 23K468-10-18 | 0 | 1 | 2 | 0 | 6.9 | 2 |
| **552** | 23K468-10-19 | 1 | 0 | 1 | 0 | 15.45 | _ |
| **553** | 23K468-10-20 | 0 | 1 | 1 | 0 | 24.59 | _ |
| **554** | 23K468-10-21 | 1 | 1 | 1 | 0 | 37.5 | 2.5 |
| **555** | 23K468-10-22 | 1 | 1 | 0 | 0 | 14.24 | _ |
| **556** | 23K468-10-23 | 1 | 0 | 2 | 0 | 16.15 | _ |
| **557** | 23K468-10-24 | 0 | 1 | 2 | 0 | 8.74 | 2 |
| **558** | 23K468-10-25 | 0 | 2 | 0 | 0 | _ | _ |
| **559** | 23K461-4-1 | 1 | 1 | 1 | 0 | 11.94 | _ |
| **560** | 23K461-4-2 | 0 | 2 | 1 | 0 | 5.44 | 3 |
| **561** | 23K461-4-3 | 1 | 2 | 0 | 0 | 34.35 | _ |
| **562** | 23K461-4-4 | 1 | 1 | 2 | 0 | 6.03 | 4.5 |
| **563** | 23K461-4-5 | 1 | 2 | 1 | 0 | _ | 3.5 |
| **564** | 23K461-4-6 | 1 | 0 | 2 | 0 | _ | _ |
| **565** | 23K461-4-7 | 0 | 1 | 2 | 0 | _ | 1.5 |
| **566** | 23K461-4-8 | 1 | 2 | 2 | 0 | _ | 1.5 |
| **567** | 23K461-4-9 | 0 | 1 | 2 | 0 | 59.04 | 2.5 |
| **568** | 23K461-4-10 | 0 | 1 | 2 | 0 | 12.69 | 2 |
| **569** | 23K461-4-11 | 1 | 0 | 0 | 0 | 36.55 | _ |
| **570** | 23K461-4-12 | 0 | 2 | 1 | 0 | _ | 2.5 |
| **571** | 23K461-4-13 | 0 | 1 | 1 | 0 | 35.71 | _ |
| **572** | 23K461-4-14 | 1 | 2 | 2 | 0 | 9.52 | 2 |
| **573** | 23K461-4-15 | 2 | 2 | 1 | 0 | 23.21 | 2 |
| **574** | 23K461-4-16 | 1 | 1 | 1 | 0 | _ | _ |
| **575** | 23K461-4-17 | 1 | 2 | 1 | 0 | 6.39 | 3.5 |
| **576** | 23K461-4-18 | 0 | 2 | 2 | 0 | _ | 3.5 |
| **577** | 23K461-4-19 | 1 | 0 | 1 | 0 | _ | _ |
| **578** | 23K461-4-20 | 1 | 1 | 0 | 0 | 7.1 | _ |
| **579** | 23K461-4-21 | 1 | 1 | 0 | 0 | _ | _ |
| **580** | 23K461-4-22 | 0 | 1 | 0 | 0 | 94.44 | _ |
| **581** | 23K461-4-23 | 1 | 0 | 1 | 0 | 11.6 | _ |
| **582** | 23K461-4-24 | 2 | 2 | 0 | 0 | 6.02 | 3.5 |
| **583** | 23K461-5-1 | 0 | 1 | 2 | 0 | 7.04 | 3 |
| **584** | 23K461-5-2 | 1 | 0 | 1 | 0 | 6.67 | _ |
| **585** | 23K461-5-3 | 1 | 1 | 1 | 0 | 16.54 | _ |
| **586** | 23K461-5-4 | 0 | 1 | 0 | 0 | _ | _ |
| **587** | 23K461-5-5 | 1 | 0 | 2 | 0 | 16.82 | 3.5 |
| **588** | 23K461-5-6 | 1 | 1 | 2 | 0 | _ | 2 |
| **589** | 23K461-5-7 | 0 | 2 | 1 | 0 | _ | 2 |
| **590** | 23K461-5-8 | 2 | 0 | 0 | 0 | _ | _ |
| **591** | 23K461-5-9 | 1 | 1 | 2 | 0 | 8.6 | 4 |
| **592** | 23K461-5-10 | 1 | 1 | 2 | 0 | _ | 4 |
| **593** | 23K461-5-11 | 2 | 2 | 1 | 0 | _ | _ |
| **594** | 23K461-5-12 | 1 | 1 | 2 | 0 | 15 | 2.5 |
| **595** | 23K461-5-13 | 2 | 0 | 2 | 0 | 7.42 | 4 |
| **596** | 23K461-5-14 | 0 | 1 | 1 | 0 | 7.52 | _ |
| **597** | 23K461-5-15 | 1 | 2 | 0 | 0 | 7.07 | 5 |
| **598** | 23K461-5-16 | 1 | 0 | 1 | 0 | 5.26 | _ |
| **599** | 23K461-5-17 | 1 | 1 | 0 | 0 | 23.53 | _ |
| **600** | 23K461-5-18 | 1 | 2 | 2 | 0 | 5.97 | 2.5 |
| **601** | 23K461-5-19 | 1 | 1 | 0 | 0 | _ | _ |
| **602** | 23K461-5-20 | 1 | 1 | 1 | 0 | 19.4 | _ |
| **603** | 23K461-5-21 | 0 | 1 | 1 | 0 | 14.4 | 2.5 |
| **604** | 23K461-5-22 | 0 | 0 | 0 | 0 | _ | _ |
| **605** | 23K461-5-23 | 1 | 1 | 2 | 0 | 5.99 | _ |
| **606** | 23K461-5-24 | 1 | 0 | 1 | 0 | _ | _ |
| **607** | 23K461-40-1 | 1 | 1 | 2 | 0 | 8.58 | 4 |
| **608** | 23K461-40-2 | 1 | 2 | 2 | 0 | 10.24 | 1.5 |
| **609** | 23K461-40-3 | 1 | 0 | 1 | 0 | 9.07 | _ |
| **610** | 23K461-40-4 | 1 | 1 | 1 | 0 | 16.25 | _ |
| **611** | 23K461-40-5 | 1 | 0 | 0 | 0 | _ | _ |
| **612** | 23K461-40-6 | 1 | 2 | 2 | 0 | _ | 3 |
| **613** | 23K461-40-7 | 0 | 2 | 2 | 0 | _ | _ |
| **614** | 23K461-40-8 | 0 | 0 | 0 | 0 | _ | _ |
| **615** | 23K461-40-9 | 1 | 1 | 1 | 0 | _ | _ |
| **616** | 23K461-40-10 | 1 | 1 | 2 | 0 | _ | 2.5 |
| **617** | 23K461-40-11 | 1 | 2 | 1 | 0 | _ | _ |
| **618** | 23K461-40-12 | 1 | 2 | 0 | 0 | _ | _ |
| **619** | 23K461-40-13 | 2 | 1 | 2 | 0 | 5.13 | 3.5 |
| **620** | 23K461-40-14 | 2 | 1 | 2 | 0 | _ | 3 |
| **621** | 23K461-40-15 | 0 | 1 | 1 | 0 | _ | _ |
| **622** | 23K461-40-16 | 0 | 2 | 2 | 0 | _ | 3 |
| **623** | 23K461-40-17 | 1 | 2 | 1 | 0 | _ | 4 |
| **624** | 23K461-40-18 | 2 | 2 | 1 | 0 | _ | 4 |
| **625** | 23K461-40-19 | 1 | 0 | 1 | 0 | 8.39 | _ |
| **626** | 23K461-40-20 | 1 | 2 | 0 | 0 | 6.87 | 3.5 |
| **627** | 23K461-40-21 | 1 | 2 | 1 | 0 | 8.03 | 3.5 |
| **628** | 23K461-40-22 | 1 | 0 | 1 | 0 | _ | _ |
| **629** | 23K461-40-23 | 2 | 0 | 0 | 0 | 6.96 | _ |
| **630** | 23K461-40-24 | 0 | 2 | 0 | 0 | 15.38 | _ |
| **631** | 23K461-40-25 | 2 | 1 | 2 | 0 | _ | 1 |
| **632** | 23K461-40-26 | 0 | 0 | 1 | 0 | _ | _ |
| **633** | 23K461-40-27 | 2 | 1 | 1 | 0 | _ | 2.5 |
| **634** | 23K461-40-28 | 0 | 1 | 0 | 0 | _ | _ |
| **635** | 23K461-40-29 | 1 | 1 | 2 | 0 | _ | 2 |
| **636** | 23K461-40-30 | 1 | 0 | 1 | 0 | _ | _ |
| **637** | 23K461-40-31 | 0 | 1 | 1 | 0 | _ | _ |
| **638** | 23K461-40-32 | 2 | 2 | 2 | 0 | _ | 2 |
| **639** | 23K461-40-33 | 0 | 2 | 1 | 0 | 6.9 | 4.5 |
| **640** | 23K461-40-34 | 0 | 1 | 2 | 0 | 5.01 | 3 |
| **641** | 23K461-40-35 | 0 | 0 | 2 | 0 | 13.28 | _ |
| **642** | 23K461-40-36 | 2 | 1 | 1 | 0 | _ | 3 |
| **643** | 23K461-40-37 | 1 | 0 | 1 | 0 | 11.67 | _ |
| **644** | 23K461-40-38 | 1 | 1 | 1 | 0 | 47.37 | _ |
| **645** | 23K461-40-39 | 0 | 0 | 2 | 0 | 10.27 | _ |
| **646** | 23K461-40-40 | 1 | 0 | 1 | 0 | _ | _ |
| **647** | 23K461-40-41 | 0 | 1 | 1 | 0 | _ | _ |
| **648** | 23K461-40-42 | 1 | 1 | 2 | 0 | 4.65 | 2.5 |
| **649** | 23K461-40-43 | 2 | 0 | 0 | 0 | 12.7 | _ |
| **650** | 23K461-40-44 | 2 | 1 | 2 | 0 | 7.07 | 3 |
| **651** | 23K461-40-45 | 1 | 1 | 1 | 0 | 7.21 | _ |
| **652** | 23K461-40-46 | 2 | 2 | 0 | 0 | _ | 3.5 |
| **653** | 23K461-40-47 | 0 | 2 | 2 | 0 | _ |  |
| **654** | 23K461-40-48 | 0 | 0 | 0 | 0 | _ | _ |
| **655** | 23K461-40-49 | 0 | 1 | 1 | 0 | 4.96 | _ |
| **656** | 23K461-40-50 | 1 | 2 | 2 | 0 | 4.88 | 3.5 |
| **657** | 23K472-49-1 | 2 | 1 | 1 | 0 | 12.77 | 2 |
| **658** | 23K472-49-2 | 1 | 0 | 1 | 0 | 36.76 | _ |
| **659** | 23K472-49-3 | 1 | 2 | 0 | 0 | 23.91 | _ |
| **660** | 23K472-49-4 | 1 | 2 | 1 | 0 | 7.62 | 4 |
| **661** | 23K472-49-5 | 1 | 1 | 0 | 0 | 7.64 | _ |
| **662** | 23K472-49-6 | 2 | 1 | 0 | 0 | _ | 4 |
| **663** | 23K472-49-7 | 2 | 1 | 0 | 0 | 11.79 | 2 |
| **664** | 23K472-49-8 | 2 | 1 | 1 | 0 | 11.37 | 2.5 |
| **665** | 23K472-49-9 | 1 | 1 | 1 | 0 | 6.07 | _ |
| **666** | 23K472-49-10 | 2 | 0 | 0 | 0 | 9.24 | _ |
| **667** | 23K472-49-11 | 0 | 1 | 1 | 0 | 26.78 | _ |
| **668** | 23K472-49-12 | 0 | 1 | 1 | 0 | 28.86 | _ |
| **669** | 23K472-49-13 | 0 | 0 | 2 | 0 | 14.85 | _ |
| **670** | 23K472-49-14 | 1 | 1 | 0 | 0 | 11.88 | _ |
| **671** | 23K472-49-15 | 0 | 0 | 2 | 0 | 4.76 | _ |
| **672** | 23K472-49-16 | 1 | 1 | 1 | 0 | 9.74 | _ |
| **673** | 23K472-49-17 | 2 | 1 | 2 | 0 | 6.68 | 2.5 |
| **674** | 23K472-49-18 | 0 | 0 | 2 | 0 | 13.36 | _ |
| **675** | 23K472-49-19 | 1 | 1 | 1 | 0 | 9.26 | _ |
| **676** | 23K472-49-20 | 1 | 2 | 2 | 0 | _ | 1.5 |
| **677** | 23K472-49-21 | 1 | 0 | 2 | 0 | 9.05 | _ |
| **678** | 23K472-49-22 | 0 | 0 | 1 | 0 | _ | _ |
| **679** | 23K472-49-23 | 0 | 2 | 0 | 0 | 33.7 | _ |
| **680** | 23K472-49-24 | 1 | 0 | 1 | 0 | 5.35 | _ |
| **681** | 23K472-50-1 | 2 | 1 | 0 | 0 | 10.24 | 2.5 |
| **682** | 23K472-50-2 | 1 | 1 | 1 | 0 | 9.38 | _ |
| **683** | 23K472-50-3 | 0 | 2 | 0 | 0 | 5.9 | _ |
| **684** | 23K472-50-4 | 0 | 1 | 1 | 0 | 70.15 | _ |
| **685** | 23K472-50-5 | 2 | 2 | 1 | 0 | 8.3 | 4 |
| **686** | 23K472-50-6 | 1 | 2 | 1 | 0 | _ | 2 |
| **687** | 23K472-50-7 | 1 | 0 | 1 | 0 | 4.76 | _ |
| **688** | 23K472-50-8 | 1 | 1 | 1 | 0 | _ | _ |
| **689** | 23K472-50-9 | 1 | 0 | 1 | 0 | 8.7 | _ |
| **690** | 23K472-50-10 | 1 | 0 | 1 | 0 | _ | _ |
| **691** | 23K472-50-11 | 1 | 1 | 0 | 0 | _ | _ |
| **692** | 23K472-50-12 | 1 | 1 | 0 | 0 | _ | _ |
| **693** | 23K472-50-13 | 1 | 0 | 2 | 0 | _ | 2.5 |
| **694** | 23K472-50-14 | 1 | 0 | 1 | 0 | 8.83 | _ |
| **695** | 23K472-50-15 | 1 | 1 | 0 | 0 | 8.28 | _ |
| **696** | 23K472-50-16 | 1 | 1 | 1 | 0 | 9.13 | _ |
| **697** | 23K472-50-17 | 2 | 1 | 1 | 0 | 5.21 | 3.5 |
| **698** | 23K472-50-18 | 2 | 0 | 1 | 0 | 5.45 | 3 |
| **699** | 23K472-50-19 | 0 | 0 | 2 | 0 | 14.53 | _ |
| **700** | 23K472-50-20 | 0 | 1 | 2 | 0 | 24.25 | 2.5 |
| **701** | 23K472-50-21 | 0 | 0 | 1 | 0 | 5 | _ |
| **702** | 23K472-50-22 | 1 | 0 | 0 | 0 | 21.69 | _ |
| **703** | 23K472-50-23 | 1 | 0 | 1 | 0 | 4.55 | _ |
| **704** | 23K472-50-24 | 2 | 2 | 1 | 0 | _ | 3 |
| **705** | 23K472-50-25 | 0 | 0 | 1 | 0 | _ | _ |
| **706** | 23K487-67-1 | 1 | 1 | 0 | 1 | 30.26 | _ |
| **707** | 23K487-67-2 | 1 | 0 | 1 | 1 | 22.34 | _ |
| **708** | 23K487-67-3 | 1 | 1 | 1 | 1 | 15.51 | _ |
| **709** | 23K487-67-4 | 0 | 0 | 0 | 2 | _ | _ |
| **710** | 23K487-67-5 | 1 | 0 | 2 | 1 | 12.19 | 3 |
| **711** | 23K487-67-6 | 2 | 2 | 1 | 1 | _ | _ |
| **712** | 23K487-67-7 | 2 | 1 | 0 | 1 | _ | 2 |
| **713** | 23K487-67-8 | 2 | 0 | 1 | 1 | _ | 4 |
| **714** | 23K487-67-9 | 1 | 1 | 0 | 1 | _ | _ |
| **715** | 23K487-67-10 | 1 | 2 | 0 | 2 | 9.31 | _ |
| **716** | 23K487-67-11 | 2 | 1 | 1 | 0 | _ | 3.5 |
| **717** | 23K487-67-12 | 1 | 1 | 2 | 0 | _ | 2 |
| **718** | 23K487-67-13 | 1 | 1 | 2 | 2 | 6.25 | 1.5 |
| **719** | 23K487-67-14 | 2 | 2 | 2 | 2 | 14.13 | 2.5 |
| **720** | 23K487-67-15 | 1 | 0 | 2 | 1 | 6.39 | 2.5 |
| **721** | 23K487-67-16 | 2 | 1 | 0 | 0 | 6.94 | 2.5 |
| **722** | 23K487-67-17 | 1 | 1 | 1 | 2 | _ | _ |
| **723** | 23K487-67-18 | 2 | 1 | 1 | 2 | _ | 4 |
| **724** | 23K487-67-19 | 0 | 1 | 2 | 1 | _ | 3 |
| **725** | 23K487-67-20 | 0 | 2 | 2 | 1 | _ | _ |
| **726** | 23K487-67-21 | 1 | 2 | 1 | 0 | _ | 3.5 |
| **727** | 23K487-67-22 | 1 | 2 | 0 | 2 | _ | 3.5 |
| **728** | 23K487-67-23 | 2 | 2 | 2 | 0 | _ | 2 |
| **729** | 23K487-67-24 | 1 | 2 | 2 | 1 | 5.98 | 3.5 |
| **730** | 23K487-67-25 | 2 | 2 | 0 | 0 | _ | 3 |
| **731** | 23K487-67-26 | 1 | 0 | 1 | 2 | 9.52 | _ |
| **732** | 23K487-67-27 | 0 | 2 | 0 | 1 | _ | _ |
| **733** | 23K487-67-28 | 0 | 0 | 0 | 1 | 18.53 | _ |
| **734** | 23K487-67-29 | 1 | 1 | 1 | 0 | 9.84 | _ |
| **735** | 23K487-67-30 | 2 | 0 | 2 | 1 | 13.75 | 3.5 |
| **736** | 23K487-67-31 | 2 | 1 | 1 | 1 | 9.36 | 3 |
| **737** | 23K487-67-32 | 2 | 0 | 2 | 2 | _ | 2 |
| **738** | 23K487-67-33 | 1 | 1 | 1 | 1 | _ | _ |
| **739** | 23K487-67-34 | 1 | 1 | 2 | 2 | 12.48 | 1.5 |
| **740** | 23K487-67-35 | 2 | 1 | 1 | 1 | _ | 3 |
| **741** | 23K487-67-36 | 1 | 1 | 1 | 1 | 7.91 | _ |
| **742** | 23K487-67-37 | 1 | 1 | 0 | 2 | _ | _ |
| **743** | 23K487-67-38 | 2 | 2 | 1 | 0 | _ | 2.5 |
| **744** | 23K487-67-39 | 2 | 1 | 1 | _ | _ | 1.5 |
| **745** | 23K487-67-40 | 1 | 1 | 1 | 2 | 26.32 | _ |
| **746** | 23K487-67-41 | 0 | 1 | 1 | 1 | 18.33 | _ |
| **747** | 23K487-67-42 | 1 | 1 | 2 | 2 | _ | 2.5 |
| **748** | 23K487-67-43 | 1 | 0 | 2 | 1 | _ | _ |
| **749** | 23K487-67-44 | 2 | 2 | 1 | 1 | _ | 4 |
| **750** | 23K487-67-45 | 2 | 1 | 0 | 1 | _ | 3 |
| **751** | 23K487-67-46 | 0 | 2 | 2 | 2 | _ | 2 |
| **752** | 23K487-67-47 | 0 | 1 | 1 | 2 | 23.53 | _ |
| **753** | 23K487-67-48 | 0 | 1 | 0 | 0 | _ | _ |
| **754** | 23K487-67-49 | 1 | 0 | 0 | 0 | 93.33 | _ |
| **755** | 23K487-67-50 | 1 | 1 | 0 | 1 | _ | _ |
| **756** | 23K466-8-1 | 1 | 0 | 0 | 0 | _ | _ |
| **757** | 23K466-8-2 | 1 | 0 | 0 | 0 | _ | _ |
| **758** | 23K466-8-3 | 1 | 0 | 0 | 0 | _ | _ |
| **759** | 23K466-8-4 | 1 | 0 | 0 | 0 | _ | _ |
| **760** | 23K466-8-5 | 2 | 0 | 0 | 0 | _ | 3.5 |
| **761** | 23K466-8-6 | 2 | 0 | 0 | 0 | _ | 3.5 |
| **762** | 23K466-8-7 | 1 | 0 | 0 | 0 | _ | _ |
| **763** | 23K466-8-8 | 1 | 0 | 0 | 0 | 52.78 | _ |
| **764** | 23K466-8-9 | 0 | 0 | 0 | 0 | _ | _ |
| **765** | 23K466-8-10 | 1 | 0 | 0 | 0 | _ | _ |
| **766** | 23K466-8-11 | 1 | 0 | 0 | 0 | _ | _ |
| **767** | 23K466-8-12 | 2 | 0 | 0 | 0 | _ | 2.5 |
| **768** | 23K466-8-13 | 1 | 0 | 0 | 0 | _ | _ |
| **769** | 23K466-8-14 | 0 | 0 | 0 | 0 | _ | _ |
| **770** | 23K466-8-15 | 1 | 0 | 0 | 0 | _ | _ |
| **771** | 23K466-8-16 | 2 | 0 | 0 | 0 | _ | _ |
| **772** | 23K466-8-17 | 0 | 0 | 0 | 0 | _ | _ |
| **773** | 23K466-8-18 | 1 | 0 | 0 | 0 | _ | _ |
| **774** | 23K466-8-19 | 0 | 0 | 0 | 0 | _ | _ |
| **775** | 23K466-8-20 | 2 | 0 | 0 | 0 | _ | _ |
| **776** | 23K466-8-21 | 0 | 0 | 0 | 0 | _ | _ |
| **777** | 23K466-8-22 | 2 | 0 | 0 | 0 | _ | 3.5 |
| **778** | 23K466-8-23 | 0 | 0 | 0 | 0 | _ | _ |
| **779** | 23K466-8-24 | 2 | 0 | 0 | 0 | _ | 3 |
| **780** | 23K466-8-25 | 2 | 0 | 0 | 0 | _ | 3 |
| **781** | 23K466-8-26 | 2 | 0 | 0 | 0 | _ | 2.5 |
| **782** | 23K466-8-27 | 0 | 0 | 0 | 0 | _ | _ |
| **783** | 23K466-8-28 | 0 | 0 | 0 | 0 | _ | _ |
| **784** | 23K466-8-29 | 0 | 0 | 0 | 0 | _ | _ |
| **785** | 23K466-8-30 | 2 | 0 | 0 | 0 | _ | _ |
| **786** | 23K466-8-31 | 0 | 0 | 0 | 0 | _ | _ |
| **787** | 23K466-8-32 | 1 | 0 | 0 | 0 | _ | _ |
| **788** | 23K466-8-33 | 1 | 0 | 0 | 0 | _ | _ |
| **789** | 23K466-8-34 | 0 | 0 | 0 | 0 | _ | _ |
| **790** | 23K466-8-35 | 1 | 0 | 0 | 0 | _ | _ |
| **791** | 23K466-8-36 | 0 | 0 | 0 | 0 | _ | _ |
| **792** | 23K466-8-37 | 1 | 0 | 0 | 0 | _ | _ |
| **793** | 23K466-8-38 | 1 | 0 | 0 | 0 | _ | _ |
| **794** | 23K466-8-39 | 0 | 0 | 0 | 0 | _ | _ |
| **795** | 23K466-8-40 | 0 | 0 | 0 | 0 | _ | _ |
| **796** | 23K466-8-41 | 1 | 0 | 0 | 0 | _ | _ |
| **797** | 23K466-8-42 | 1 | 0 | 0 | 0 | _ | _ |
| **798** | 23K466-8-43 | 1 | 0 | 0 | 0 | _ | _ |
| **799** | 23K466-8-44 | 1 | 0 | 0 | 0 | _ | _ |
| **800** | 23K466-8-45 | 1 | 0 | 0 | 0 | _ | _ |
| **801** | 23K466-8-46 | 1 | 0 | 0 | 0 | _ | _ |
| **802** | 23K466-8-47 | 1 | 0 | 0 | 0 | _ | _ |
| **803** | 23K466-8-48 | 0 | 0 | 0 | 0 | _ | _ |
| **804** | 23K466-8-49 | 0 | 0 | 0 | 0 | _ | _ |
| **805** | 23K466-8-50 | 1 | 0 | 0 | 0 | _ | _ |
| **806** | 23K478-19-1 | 1 | 0 | 0 | 0 | _ | _ |
| **807** | 23K478-19-2 | 2 | 0 | 0 | 0 | _ | 3.5 |
| **808** | 23K478-19-3 | 2 | 0 | 0 | 0 | _ | 3.5 |
| **809** | 23K478-19-4 | 1 | 0 | 0 | 0 | _ | _ |
| **810** | 23K478-19-5 | 1 | 0 | 0 | 0 | _ | _ |
| **811** | 23K478-19-6 | 2 | 0 | 0 | 0 | _ | 4.5 |
| **812** | 23K478-19-7 | 1 | 0 | 0 | 0 | _ | 4 |
| **813** | 23K478-19-8 | 1 | 0 | 0 | 0 | _ | 3.5 |
| **814** | 23K478-19-9 | 0 | 0 | 0 | 0 | _ | _ |
| **815** | 23K478-19-10 | 2 | 0 | 0 | 0 | 12.84 | 2.5 |
| **816** | 23K478-19-11 | 1 | 0 | 0 | 0 | _ | 1.5 |
| **817** | 23K478-19-12 | 1 | 0 | 0 | 0 | _ | 1 |
| **818** | 23K478-19-13 | 2 | 0 | 0 | 0 | _ | _ |
| **819** | 23K478-19-14 | 2 | 0 | 0 | 0 | 10 | 4 |
| **820** | 23K478-19-15 | 0 | 0 | 0 | 0 | _ | _ |
| **821** | 23K478-19-16 | 2 | 0 | 0 | 0 | _ | 1.5 (dwarf) |
| **822** | 23K478-19-17 | 1 | 0 | 0 | 0 | _ | 2 |
| **823** | 23K478-19-18 | 1 | 0 | 0 | 0 | _ | 3 |
| **824** | 23K478-19-19 | 1 | 0 | 0 | 0 | 14.62 | 3.5 |
| **825** | 23K478-19-20 | 2 | 0 | 0 | 0 | 9.41 | 3.5 |
| **826** | 23K478-19-21 | 1 | 0 | 0 | 0 | 16.67 | 3.5 |
| **827** | 23K478-19-22 | 0 | 0 | 0 | 0 | _ | _ |
| **828** | 23K478-19-23 | 1 | 0 | 0 | 0 | _ | _ |
| **829** | 23K478-19-24 | 2 | 0 | 0 | 0 | _ | 1 |
| **830** | 23K478-19-25 | 1 | 0 | 0 | 0 | _ | 3 |
| **831** | 23K478-19-26 | 2 | 0 | 0 | 0 | _ | _ |
| **832** | 23K478-19-27 | 0 | 0 | 0 | 0 | _ | _ |
| **833** | 23K478-19-28 | 2 | 0 | 0 | 0 | _ | 3 (dwarf) |
| **834** | 23K478-19-29 | 0 | 0 | 0 | 0 | 25 | _ |
| **835** | 23K478-19-30 | 2 | 0 | 0 | 0 | _ | _ |
| **836** | 23K478-19-31 | 2 | 0 | 0 | 0 | 72.22 | 2 |
| **837** | 23K478-19-32 | 1 | 0 | 0 | 0 | 13.05 | 2 |
| **838** | 23K478-19-33 | 0 | 0 | 0 | 0 | _ | _ |
| **839** | 23K478-19-34 | 1 | 0 | 0 | 0 | 7.69 | 2.5 |
| **840** | 23K478-19-35 | 0 | 0 | 0 | 0 | _ | 4.5 |
| **841** | 23K478-19-36 | 1 | 0 | 0 | 0 | _ | 4.5 |
| **842** | 23K478-19-37 | 0 | 0 | 0 | 0 | _ | _ |
| **843** | 23K478-19-38 | 1 | 0 | 0 | 0 | _ | 2.5 |
| **844** | 23K478-19-39 | 1 | 0 | 0 | 0 | _ | 2.5 |
| **845** | 23K478-19-40 | 1 | 0 | 0 | 0 | _ | 4.5 |
| **846** | 23K478-19-41 | 1 | 0 | 0 | 0 | 22.71 | _ |
| **847** | 23K478-19-42 | 1 | 0 | 0 | 0 | 17.25 | 4 |
| **848** | 23K478-19-43 | 2 | 0 | 0 | 0 | _ | _ |
| **849** | 23K478-19-44 | 0 | 0 | 0 | 0 | _ | _ |
| **850** | 23K478-19-45 | 2 | 0 | 0 | 0 | _ | 2 |
| **851** | 23K478-19-46 | 2 | 0 | 0 | 0 | _ | 3 |
| **852** | 23K478-19-47 | 1 | 0 | 0 | 0 | _ | _ |
| **853** | 23K478-19-48 | 1 | 0 | 0 | 0 | _ | _ |
| **854** | 23K478-19-49 | 1 | 0 | 0 | 0 | _ | 2 |
| **855** | 23K478-19-50 | 0 | 0 | 0 | 0 | _ | _ |

*^1^ Qfhb.rwg-5A.1*; ^2^ *Qfhb.rwg-5A.2*; ^3^ Average Disease Severity; ^4^ Agronomic score
