# Supplementary material for: Pre-breeding to establish Fhb7 in winter wheat using marker-assisted, modified backcrossing
Source: Front Plant Sci. 2026 Aug 26;17:1863042. doi: 10.3389/fpls.2026.1863042 (PMC13553846; doi:10.3389/fpls.2026.1863042)
Supplement: Supplementary file 2 [file Table2.docx]

**Supplementary Table 2.** Summary of the FHB resistance gene markers that were detected in 94 plant selections. Only plants with marker pyramids that included the *Fhb7* markers were retained. FHB DS data were not generated for this set. Resistance gene marker alleles that occurred in the homozygous state are underlined; those that were heterozygous are bracketed and those without the critical marker allele are indicated with a dash.

| **S.N.** | **Cross^1^** | ***Fhb7*** | ***5a.1^2^*** | ***5a.2^2^*** | ***Fhb1*** | **Sp^2^** | **Days^2^** | **Ht^2^** | **S/S^2^** | **Set^2^** | **100K^2^** | **In^2^** |
| --- | --- | --- | --- | --- | --- | --- | --- | --- | --- | --- | --- | --- |
| 1 | C | *Fhb7* | *5a.1* | *[5a.2]* | *Fhb1* | 9 | 115 | 84 | 14 | 77 | 3.35 | 323 |
| 2 | C | *Fhb7* | *5a.1* | *[5a.2]* | *Fhb1* | 10 | 115 | 88 | 12 | 83 | 3.56 | 356 |
| 3 | C | *Fhb7* | *5a.1* | *[5a.2]* | *Fhb1* | 8 | 116 | 79 | 12 | 83 | 3.72 | 298 |
| 4 | C | *Fhb7* | *5a.1* | *[5a.2]* | *Fhb1* | 7 | 116 | 80 | 14 | 64 | 3.86 | 243 |
| 5 | C | *Fhb7* | *5a.1* | *[5a.2]* | *Fhb1* | 11 | 116 | 80 | 14 | 46 | 3.39 | 242 |
| 6 | C | *Fhb7* | *5a.1* | *[5a.2]* | *Fhb1* | 6 | 117 | 90 | 14 | 82 | 3.21 | 222 |
| 7 | C | *Fhb7* | *5a.1* | *[5a.2]* | *Fhb1* | 8 | 115 | 81 | 14 | 50 | 3.95 | 221 |
| 8 | C | *Fhb7* | *5a.1* | *_* | *Fhb1* | 5 | 117 | 85 | 14 | 75 | 3.15 | 165 |
| 9 | C | *Fhb7* | *[5a.1]* | *[5a.2]* | *Fhb1* | 4 | 119 | 75 | 14 | 82 | 3.81 | 174.95 |
| 10 | C | *Fhb7* | *[5a.1]* | *[5a.2]* | *Fhb1* | 8 | 118 | 70 | 18 | 89 | 3.21 | 411 |
| 11 | C | *Fhb7* | *[5a.1]* | *[5a.2]* | *Fhb1* | 7 | 118 | 88 | 18 | 72 | 3.63 | 331 |
| 12 | C | *Fhb7* | *[5a.1]* | *[5a.2]* | *Fhb1* | 6 | 118 | 84 | 18 | 92 | 3.26 | 323 |
| 13 | C | *Fhb7* | *[5a.1]* | *[5a.2]* | *Fhb1* | 7 | 120 | 86 | 16 | 72 | 3.91 | 315 |
| 14 | C | *Fhb7* | *[5a.1]* | *[5a.2]* | *Fhb1* | 9 | 117 | 88 | 14 | 93 | 3.18 | 372 |
| 15 | C | *Fhb7* | *[5a.1]* | *[5a.2]* | *Fhb1* | 4 | 117 | 84 | 12 | 104 | 4.58 | 229 |
| 16 | C | *Fhb7* | *[5a.1]* | *[5a.2]* | *Fhb1* | 6 | 120 | 84 | 16 | 100 | 3.11 | 299 |
| 17 | C | *Fhb7* | *[5a.1]* | *[5a.2]* | *Fhb1* | 7 | 118 | 76 | 16 | 69 | 3.34 | 257 |
| 18 | C | *Fhb7* | *[5a.1]* | *[5a.2]* | *Fhb1* | 5 | 120 | 83 | 14 | 100 | 3.63 | 254 |
| 19 | C | *Fhb7* | *[5a.1]* | *[5a.2]* | *Fhb1* | 4 | 116 | 85 | 14 | 82 | 3.77 | 174 |
| 20 | C | *Fhb7* | *[5a.1]* | *[5a.2]* | *Fhb1* | 7 | 120 | 85 | 16 | 59 | 2.58 | 171 |
| 21 | C | *Fhb7* | *[5a.1]* | *[5a.2]* | *Fhb1* | 4 | 118 | 76 | 14 | 82 | 3.71 | 171 |
| 22 | C | *Fhb7* | *[5a.1]* | *[5a.2]* | *Fhb1* | 5 | 120 | 81 | 14 | 64 | 3.3 | 148 |
| 23 | C | *Fhb7* | *[5a.1]* | *[5a.2]* | *Fhb1* | 8 | 119 | 89 | 14 | 75 | 3.4 | 286 |
| 24 | C | *Fhb7* | _ | *[5a.2]* | *Fhb1* | 9 | 114 | 75 | 14 | 79 | 3.56 | 353 |
| 25 | C | *Fhb7* | *_* | *[5a.2]* | *Fhb1* | 7 | 115 | 89 | 14 | 82 | 4.16 | 335 |
| 26 | C | *Fhb7* | *_* | *[5a.2]* | *Fhb1* | 10 | 114 | 85 | 12 | 92 | 4.06 | 447 |
| 27 | C | *Fhb7* | *_* | *[5a.2]* | *Fhb1* | 6 | 120 | 85 | 14 | 93 | 3.87 | 302 |
| 28 | C | *Fhb7* | *_* | *[5a.2]* | *Fhb1* | 3 | 120 | 87 | 20 | 63 | 3.7 | 139 |
| 29 | C | *Fhb7* | *_* | *[5a.2]* | *Fhb1* | 4 | 119 | 84 | 14 | 64 | 3.38 | 122 |
| 30 | C | *Fhb7* | *_* | *[5a.2]* | *Fhb1* | 5 | 118 | 83 | 14 | 93 | 4.09 | 266 |
| 31 | C | *Fhb7* | *_* | *[5a.2]* | *Fhb1* | 5 | 120 | 85 | 16 | 81 | 4.06 | 264 |
| 32 | C | *Fhb7* | *_* | *[5a.2]* | *Fhb1* | 4 | 119 | 76 | 16 | 75 | 3.8 | 182 |
| 33 | C | *Fhb7* | *[5a.1]* | *_* | *Fhb1* | 10 | 114 | 80 | 14 | 82 | 3.79 | 436 |
| 34 | C | *Fhb7* | *[5a.1]* | *_* | *Fhb1* | 6 | 117 | 90 | 16 | 100 | 3.36 | 322 |
| 35 | C | *Fhb7* | *[5a.1]* | *_* | *Fhb1* | 6 | 119 | 81 | 16 | 69 | 3.47 | 229 |
| 36 | C | *Fhb7* | *[5a.1]* | *_* | *Fhb1* | 6 | 116 | 76 | 14 | 71 | 4.59 | 275 |
| 37 | C | *Fhb7* | *[5a.1]* | *_* | *Fhb1* | 4 | 119 | 87 | 16 | 69 | 3.95 | 174 |
| 38 | C | *Fhb7* | *_* | *_* | *Fhb1* | 10 | 115 | 89 | 14 | 82 | 3.89 | 447 |
| 39 | C | *Fhb7* | *5a.1* | *[5a.2]* | [*Fhb1*] | 7 | 115 | 81 | 12 | 92 | 4 | 308 |
| 40 | C | *Fhb7* | *5a.1* | *[5a.2]* | [*Fhb1*] | 8 | 115 | 84 | 14 | 75 | 3.96 | 333 |
| 41 | C | *Fhb7* | *5a.1* | *[5a.2]* | [*Fhb1*] | 7 | 116 | 81 | 14 | 79 | 3.62 | 278 |
| 42 | C | *Fhb7* | *5a.1* | *[5a.2]* | [*Fhb1*] | 4 | 117 | 80 | 14 | 75 | 3.71 | 156 |
| 43 | C | *Fhb7* | *5a.1* | *[5a.2]* | [*Fhb1*] | 5 | 117 | 87 | 14 | 68 | 2.82 | 134 |
| 44 | C | *Fhb7* | *5a.1* | *[5a.2]* | [*Fhb1*] | 4 | 118 | 83 | 12 | 67 | 3.87 | 124 |
| 45 | C | *Fhb7* | *5a.1* | *[5a.2]* | [*Fhb1*] | 5 | 117 | 82 | 12 | 92 | 3.43 | 188 |
| 46 | C | *Fhb7* | *5a.1* | *[5a.2]* | [*Fhb1*] | 9 | 117 | 82 | 12 | 54 | 3.22 | 188 |
| 47 | C | *Fhb7* | *5a.1* | *_* | [*Fhb1*] | 7 | 115 | 83 | 12 | 83 | 3.49 | 244 |
| 48 | C | *Fhb7* | *5a.1* | *_* | [*Fhb1*] | 7 | 115 | 87 | 14 | 68 | 3.24 | 216 |
| 49 | C | *Fhb7* | *5a.1* | *_* | [*Fhb1*] | 6 | 117 | 81 | 10 | 100 | 3.45 | 207 |
| 50 | B | *Fhb7* | *5a.1* | *[5a.2]* | _ | 9 | 120 | 85 | 14 | 93 | 3.62 | 424 |
| 51 | B | *Fhb7* | *5a.1* | *[5a.2]* | _ | 8 | 120 | 88 | 14 | 93 | 3.98 | 414 |
| 52 | B | *Fhb7* | *5a.1* | *[5a.2]* | _ | 7 | 120 | 89 | 14 | 96 | 4.13 | 390 |
| 53 | B | *Fhb7* | *5a.1* | *[5a.2]* | _ | 6 | 119 | 86 | 16 | 84 | 4.13 | 334 |
| 54 | C | *Fhb7* | *5a.1* | *[5a.2]* | _ | 10 | 117 | 86 | 14 | 71 | 3.39 | 339 |
| 55 | C | *Fhb7* | *5a.1* | *[5a.2]* | _ | 6 | 115 | 85 | 14 | 100 | 3.66 | 308 |
| 56 | B | *Fhb7* | *5a.1* | *[5a.2]* | _ | 6 | 119 | 86 | 16 | 88 | 3.71 | 311 |
| 57 | B | *Fhb7* | *5a.1* | *[5a.2]* | _ | 7 | 115 | 88 | 14 | 82 | 3.98 | 320 |
| 58 | B | *Fhb7* | *5a.1* | *[5a.2]* | _ | 6 | 119 | 70 | 16 | 75 | 4 | 288 |
| 59 | B | *Fhb7* | *5a.1* | *[5a.2]* | _ | 5 | 119 | 90 | 20 | 80 | 3.6 | 288 |
| 60 | B | *Fhb7* | *5a.1* | *[5a.2]* | _ | 5 | 120 | 89 | 14 | 96 | 3.97 | 268 |
| 61 | B | *Fhb7* | *5a.1* | *[5a.2]* | _ | 5 | 122 | 81 | 18 | 75 | 3.62 | 245 |
| 62 | C | *Fhb7* | *5a.1* | *[5a.2]* | _ | 7 | 116 | 74 | 14 | 57 | 3.83 | 215 |
| 63 | B | *Fhb7* | *5a.1* | *[5a.2]* | _ | 3 | 120 | 77 | 14 | 75 | 3.88 | 122 |
| 64 | B | *Fhb7* | *5a.1* | *[5a.2]* | _ | 2 | 118 | 77 | 16 | 81 | 4.11 | 107 |
| 65 | B | *Fhb7* | *5a.1* | *[5a.2]* | _ | 4 | 118 | 70 | 18 | 61 | 4.3 | 189 |
| 66 | B | *Fhb7* | *5a.1* | *[5a.2]* | _ | 5 | 118 | 88 | 16 | 59 | 4.2 | 200 |
| 67 | C | *Fhb7* | *5a.1* | *_* | _ | 10 | 119 | 88 | 14 | 86 | 3.8 | 456 |
| 68 | B | *Fhb7* | *5a.1* | *_* | _ | 5 | 121 | 90 | 18 | 83 | 4.23 | 317 |
| 69 | B | *Fhb7* | *5a.1* | *_* | _ | 6 | 118 | 83 | 16 | 81 | 3.96 | 309 |
| 70 | C | *Fhb7* | *5a.1* | *_* | _ | 8 | 117 | 85 | 12 | 71 | 3.63 | 247 |
| 71 | B | *Fhb7* | *5a.1* | *_* | _ | 5 | 120 | 80 | 14 | 86 | 3.96 | 237 |
| 72 | B | *Fhb7* | *5a.1* | *_* | _ | 4 | 119 | 82 | 18 | 75 | 4.11 | 222 |
| 73 | C | *Fhb7* | *5a.1* | *_* | _ | 5 | 115 | 88 | 12 | 88 | 4.24 | 223 |
| 74 | C | *Fhb7* | *5a.1* | *_* | _ | 6 | 116 | 75 | 10 | 95 | 3.41 | 194 |
| 75 | C | *Fhb7* | *5a.1* | *_* | _ | 6 | 117 | 90 | 16 | 59 | 3.37 | 192 |
| 76 | C | *Fhb7* | *_* | *[5a.2]* | [*Fhb1*] | 12 | 120 | 90 | 20 | 95 | 4.06 | 926 |
| 77 | C | *Fhb7* | *_* | *[5a.2]* | [*Fhb1*] | 6 | 120 | 85 | 8 | 200 | 4.85 | 466 |
| 78 | C | *Fhb7* | *_* | *[5a.2]* | [*Fhb1*] | 8 | 115 | 84 | 16 | 88 | 3.13 | 351 |
| 79 | C | *Fhb7* | *_* | *[5a.2]* | [*Fhb1*] | 8 | 118 | 73 | 16 | 81 | 3.55 | 369 |
| 80 | C | *Fhb7* | *_* | *[5a.2]* | [*Fhb1*] | 3 | 120 | 77 | 20 | 88 | 2.33 | 123 |
| 81 | C | *Fhb7* | *_* | *[5a.2]* | [*Fhb1*] | 5 | 118 | 88 | 16 | 91 | 4.12 | 299 |
| 82 | B | *Fhb7* | *[5a.1]* | *[5a.2]* | _ | 4 | 122 | 82 | 16 | 78 | 3.7 | 185 |
| 83 | B | *Fhb7* | *[5a.1]* | *[5a.2]* | _ | 6 | 130 | 86 | 14 | 68 | 3.78 | 215 |
| 84 | B | *Fhb7* | *[5a.1]* | *[5a.2]* | _ | 5 | 122 | 79 | 14 | 93 | 3.41 | 221 |
| 85 | B | *Fhb7* | *[5a.1]* | *_* | _ | 7 | 122 | 83 | 18 | 92 | 3.91 | 451 |
| 86 | B | *Fhb7* | *_* | *[5a.2]* | _ | 8 | 121 | 80 | 16 | 100 | 3.9 | 499 |
| 87 | C | *Fhb7* | *_* | *[5a.2]* | _ | 6 | 125 | 75 | 20 | 65 | 3.42 | 267 |
| 88 | B | *Fhb7* | *_* | *[5a.2]* | _ | 4 | 122 | 87 | 18 | 83 | 3.38 | 203 |
| 89 | C | *Fhb7* | *_* | *[5a.2]* | _ | 5 | 117 | 86 | 16 | 88 | 3.95 | 277 |
| 90 | C | *Fhb7* | *_* | *_* | [*Fhb1*] | 5 | 119 | 88 | 16 | 81 | 4.11 | 267 |
| 91 | A | [*Fhb7*] | *[5a.1]* | *5a.2* | _ | 9 | 115 | 88 | 16 | 119 | 4.47 | 764 |
| 92 | A | [*Fhb7*] | *[5a.1]* | *5a.2* | _ | 13 | 117 | 86 | 14 | 86 | 3.79 | 591 |
| 93 | A | [*Fhb7*] | *[5a.1]* | *5a.2* | _ | 5 | 117 | 83 | 16 | 100 | 4.57 | 366 |
| 94 | A | [*Fhb7*] | *[5a.1]* | *5a.2* | _ | 3 | 116 | 87 | 14 | 86 | 3.97 | 143 |

¹ See Supplementary Table 3.
^2^ Abbreviations used: *5a.1* (*Qfhb.rwg-5A.1*); *5a.2* (*Qfhb.rwg-5A.2*); Sp (number of spikelets); day (days to heading); Ht (plant height_cm); S/S (spikelets/spike); Set (seed set %); 100K (100 kernel weight in grams); In (index number)
