# Supplementary material for: Pre-breeding to establish Fhb7 in winter wheat using marker-assisted, modified backcrossing
Source: Front Plant Sci. 2026 Aug 26;17:1863042. doi: 10.3389/fpls.2026.1863042 (PMC13553846; doi:10.3389/fpls.2026.1863042)
Supplement: Supplementary file 3 [file Table3.docx]

| **Marker** | **Genotype** | **Vernalized (n)** | **Non-vernalized (n)** | **χ²** | **df** | **p-value** |
| --- | --- | --- | --- | --- | --- | --- |
| *Fhb7* |  | (145) | (177) |  |  |  |
|  | AA | 28(19.3%) | 46(26.0%) |  |  |  |
|  | Aa | 84(57.9%) | 80(45.2%) | 5.20 | 2 | 0.074 |
|  | aa | 33(22.8%) | 51(28.8%) |  |  |  |
| *Qfhb.rwg-5A-1* |  | (133) | (89) |  |  |  |
|  | AA | 25(18.8%) | 30(33.7%) |  |  |  |
|  | Aa | 67(50.4%) | 39(43.8%) | 6.62 | 2 | 0.037* |
|  | aa | 41(30.8%) | 20(22.5%) |  |  |  |
| *Qfhb.rwg-5A-2* |  | (133) | (89) |  |  |  |
|  | AA | 41(30.8%) | 25(28.1%) |  |  |  |
|  | Aa | 62(46.6%) | 38(42.7%) | 1.25 | 2 | 0.534 |
|  | aa | 30(22.6%) | 26(29.2%) |  |  |  |
| *Fhb1* |  | (28) | (21) |  |  |  |
|  | AA | 8(28.6%) | 7(33.3%) |  |  |  |
|  | Aa | 13(46.4%) | 11(52.4%) | 0.85 | 2 | 0.654 |
|  | aa | 7(25.0%) | 3(14.3%) |  |  |  |

**Supplementary Table 3.** Comparison of marker genotype distributions between fully vernalized and non-vernalized BC_3_F_2_ plants

n = group sample size; percentages are within-group. χ² = chi-square test of independence; df = degrees of freedom. *p < 0.05.
