# Supplementary material for: Pre-breeding to establish Fhb7 in winter wheat using marker-assisted, modified backcrossing
Source: Front Plant Sci. 2026 Aug 26;17:1863042. doi: 10.3389/fpls.2026.1863042 (PMC13553846; doi:10.3389/fpls.2026.1863042)
Supplement: Supplementary file 4 [file Table4.docx]

**Supplementary table 4.** Goodness-of-fit tests for expected 1:2:1 Mendelian segregation ratios among fully vernalized BC₃F₂ plants.

| **Marker** | **Genotype** | **Observed** | **Expected (1:2:1)** | **χ²** | **df** | **p-value** |
| --- | --- | --- | --- | --- | --- | --- |
| *Fhb7* | AA | 28 | 36.25 |  |  |  |
|  | Aa | 84 | 72.5 | 3.99 | 2 | 0.14 |
|  | Aa | 33 | 36.25 |  |  |  |
| *Qfhb.rwg-5A-1* | AA | 25 | 33.25 |  |  |  |
|  | Aa | 67 | 66.5 | 3.86 | 2 | 0.14 |
|  | Aa | 41 | 33.25 |  |  |  |
| *Qfhb.rwg-5A-2* | AA | 41 | 33.25 |  |  |  |
|  | Aa | 62 | 66.5 | 2.43 | 2 | 0.3 |
|  | aa | 30 | 33.25 |  |  |  |
| *Fhb1* | AA | 8 | 7 |  |  |  |
|  | Aa | 13 | 14 | 0.21 | 2 | 0.9 |
|  | aa | 7 | 7 |  |  |  |

χ² = chi-square goodness-of-fit statistic (observed vs. expected 1:2:1); df = degrees of freedom. *Significant deviation from the expected 1:2:1 ratio at p < 0.05. No marker showed a significant deviation in this analysis.
